# Ternary Molecular Co‐Assembling Heterogeneous Membranes for High‐Efficiency and Anti‐Biofouling Osmotic Energy Conversion

**DOI:** 10.1002/advs.202504843

**Published:** 2025-07-13

**Authors:** Minmin Li, Yuting Xiong, Fenglin Zhang, Haijie Wei, Yuchen Cao, Guangyan Qing

**Affiliations:** ^1^ State Key Laboratory of Medical Proteomics Dalian Institute of Chemical Physics Chinese Academy of Sciences Dalian 116023 P. R. China; ^2^ Dalian Lingshui Bay Laboratory Dalian 116023 P. R. China

**Keywords:** anti‐biofouling, asymmetric nanochannels, heterogeneous membranes, molecular co‐assembly, osmotic energy conversion

## Abstract

Low‐cost access to abundant, green osmotic energy through nanofluidic membranes holds promise to solve the economic, energy, and environmental trilemma. Despite considerable promising explorations, developing a nanofluidic membrane with ease of fabrication, high performance, and superior biofouling resistance remains a challenge. Here, a unique ternary molecular co‐assembly strategy is presented to provide a solution. L‐glutathione, Ag^+^ ions, and histamine can co‐assemble into twisted nanofibers through coordination and cross‐linking. The co‐assembly within anodic aluminum oxide (AAO) nanochannels, reinforced by polyvinyl alcohol (PVA), creates a heterogeneous membrane (AAO@GHAg/PVA) characterized by enhanced stability and mechanical strength. The AAO@GHAg/PVA membrane features asymmetric nanochannels that facilitate anion‐selective unidirectional transport, achieving a peak output power density of 17.5 W·m^−2^ under a 50‐fold salinity gradient. The inclusion of Ag endows AAO@GHAg/PVA with superior biofouling resistance, allowing it to maintain a maximum power density of 16.6 W·m^−2^ in a real seawater/river water environment. Interestingly, replacing histamine with spermine further boosts performance to 21.4 W·m^−2^, demonstrating the extensibility of the molecular precursors. This work opens up a new avenue for the facile construction of high‐efficiency nanofluidic membranes for osmotic energy harvesting.

## Graphical abstract

A new strategy based on ternary molecular co‐assembly for the facile construction of nanofluidic membranes is presented. The membrane exhibits asymmetric nanochannels that facilitate anion‐selective transport, achieving an osmotic energy harvesting performance of 16.6 W·m^−2^ and a superior anti‐biofouling activity in natural waters. This strategy demonstrates the extensibility of molecule precursors and the potential for further optimization and improvement.

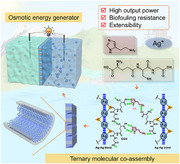

## 1 Introduction

Osmotic energy derived from the salinity difference between seawater and river water is a vast, sustainable, and clean energy source.^[^ ^1^ ^]^ Harvesting and exploiting osmotic energy hold promise for solving severe environmental issues and energy crises. Reverse electrodialysis (RED) based on ion exchange membranes provides a viable technique for the direct conversion of osmotic energy into electricity.^[^ ^2^ ^]^ However, for decades, conventional ion exchange membranes for RED have suffered from persistent intrinsic drawbacks: high membrane resistance and low ion transfer throughput.^[^ ^3^ ^]^ In recent years, with the rapid development of materials science, novel nanofluidic membranes that feature abundant nano‐/subnano‐sized channels and enriched space charges have continued to emerge, representing a technological breakthrough in improving osmotic energy harvesting performance.^[^ ^4^, ^5^, ^6^, ^7^ ^]^ In the continuous pursuit of high ion selectivity and permeability, a variety of engineered nanofluidic membranes derived from various materials, including 1D nanofibers,^[^ ^8^, ^9^, ^10^ ^]^ 2D materials,^[^ ^11^, ^12^, ^13^, ^14^, ^15^ ^]^ 3D polymers or hydrogels,^[^ ^16^, ^17^, ^18^, ^19^ ^]^ and crystalline porous materials,^[^ ^20^, ^21^, ^22^, ^23^, ^24^, ^25^ ^]^ have been explored. Despite the potential for practical applications, most of them still suffer from limited performance and cumbersome precursor synthesis processes. Moreover, the vast majority of them are not designed to resist biofouling, despite the fact that biofouling can be a serious issue, causing performance degradation in natural water environments.^[^ ^26^ ^]^ Therefore, there is an urgent need to develop nanofluidic membranes featuring simple fabrication, enhanced biofouling resistance, and high performance in natural water environments.

Self‐assembly is a ubiquitous process in nature where pre‐existing disordered components form an organized structure or motif, providing a straightforward approach to fabricate materials with tailored properties and functions.^[^ ^27^ ^]^ To date, self‐assembly techniques have been explored to construct nanofluidic membranes using diverse building blocks, such as polymers,^[^ ^28^, ^29^, ^30^ ^]^ polyoxometalates,^[^ ^31^ ^]^ metal‐organic frameworks (MOFs),^[^ ^32^, ^33^ ^]^ 2D nanosheets,^[^ ^34^, ^35^ ^]^ and cellulose nanocrystals.^[^ ^36^, ^37^ ^]^ Recently, our group also demonstrated highly efficient osmotic energy harvesting through a heterogeneous MOF membrane prepared via water‐air interfacial self‐assembly of the polymer‐modified UiO‐66‐NH_2_ nanoparticles.^[^ ^32^ ^]^ However, these assembly systems face two inherent limitations: 1) the often complicated synthesis, extraction, or exfoliation processes required for their building blocks partially negate the advantages of self‐assembly, and 2) the reliance on larger building blocks restricts the formation of precisely controlled nanoporous architectures. Molecular self‐assembly based on small molecules as simple building blocks is a true implementation of the bottom‐up assembly strategy, analogous to the formation of the cell membrane via phospholipids.^[^ ^38^ ^]^ However, to the best of our knowledge, there has been no research on the production of nanofluidic membranes through molecular self‐assembly. The main challenges include: 1) it is difficult for small molecules to self‐assemble into stable membranes with high mechanical strength and durability suitable for application in natural water environments; 2) a nanofluidic membrane must have abundant nano‐ or even subnano‐sized channels and enriched space charges, which poses high demands on the molecular self‐assembly system; 3) the typical unimolecular self‐assembly systems usually lack sufficient chemical and functional diversity, making it difficult to meet the multifaceted needs of nanofluidic membranes.

**Scheme 1:**
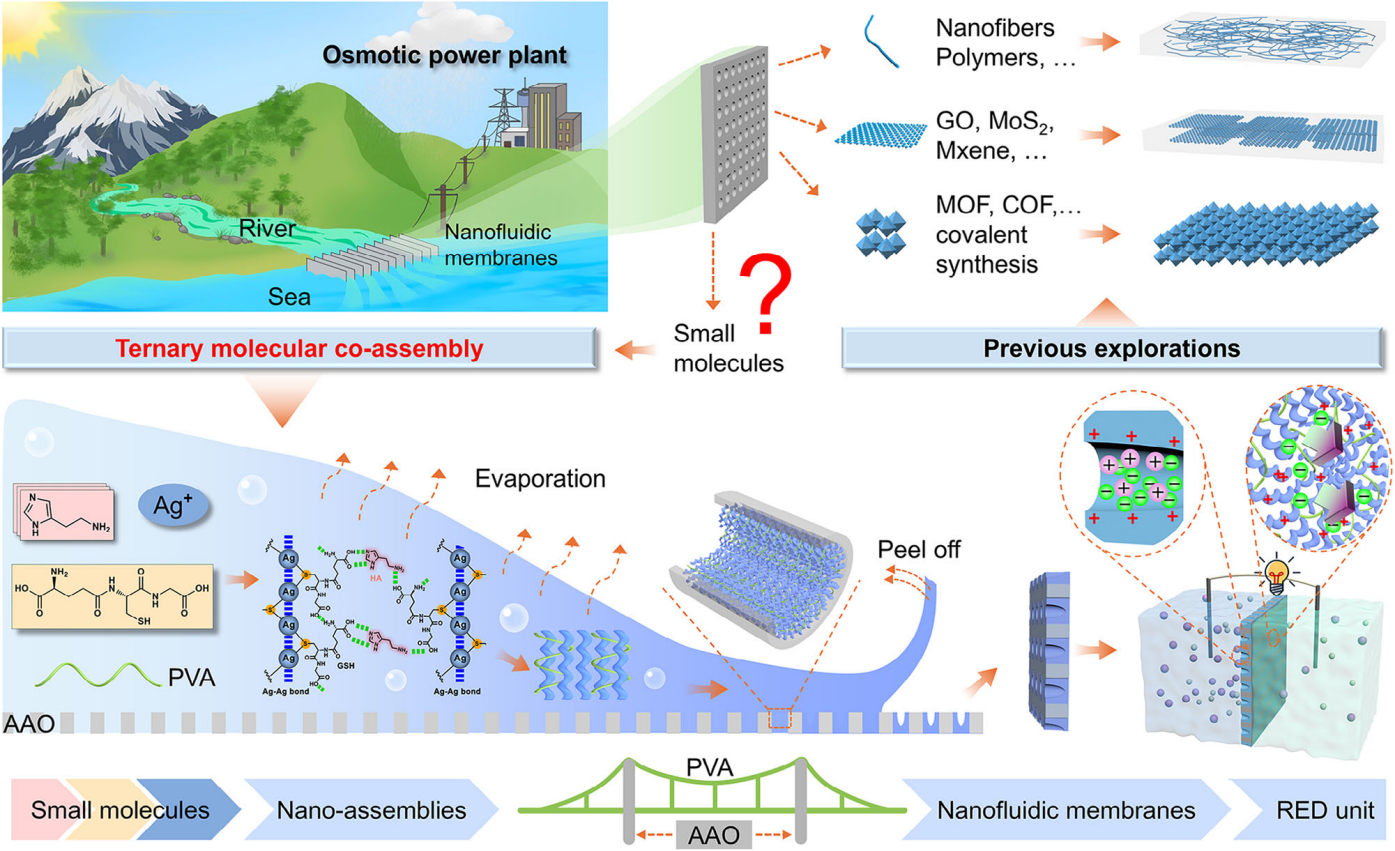
Schematic illustration of the molecular co‐assembly strategy for constructing a nanofluidic membrane. The top right shows previous designs that primarily relied on 1D nanowires, 2D nanoflakes, 3D framework materials, and other bulk materials. The bottom part depicts the ternary molecular co‐assembly strategy based on simple small molecules explored in this work.

Co‐assembly of multi‐component small molecules can fully combine their respective properties and fabricate macroscopic materials with desired functionalities and improved physicochemical characteristics. This approach provides a potential solution to fill the gap. In this work, we present a unique three‐component molecular co‐assembly strategy that enables the construction of heterogeneous nanofluidic membranes for high‐efficiency osmotic energy harvesting (**Scheme** **1**). L‐glutathione (GSH) and Ag^+^ ions can coordinate and co‐assemble into twisted nanofibers. Histamine (HA) participates in and facilitates the co‐assembly by enabling nanofiber cross‐linking through hydrogen bonds and/or electrostatic forces. Ternary co‐assembly of GSH, Ag^+^ ions, and HA within anodic aluminum oxide (AAO) nanochannels with the reinforcement of polyvinyl alcohol (PVA) yields a heterogeneous membrane, AAO@GHAg/PVA. Here, AAO acts as a substrate that supports the creation of nanoconfined spaces either between adjacent nano‐assemblies or between assemblies and channel walls, thereby enhancing ion permselectivity, while PVA intertwines with the nano‐assemblies to improve the mechanical strength and stability of the heterogeneous membrane. The AAO@GHAg/PVA membrane features structurally asymmetric nanochannels with enriched positive surface charges, exhibiting distinct diode‐like anion‐selective transport characteristics. As a result, the AAO@GHAg/PVA membrane achieves a peak output power density of 17.5 W·m^−2^ under a 50‐fold salinity gradient. Notably, the presence of Ag^+^ ions endows AAO@GHAg/PVA with superior anti‐biofouling activity. More interestingly, the HA components can be generalized to other polyamine analogues. For example, using spermine to co‐assemble with GSH and Ag^+^ ions improves the performance to 21.4 W·m^−2^, demonstrating the extensibility of the co‐assembly precursor and the potential for further optimization. This work provides a new approach for the construction of high‐performance nanofluidic membranes and establishes a paradigm for the development of membrane materials based on a self‐assembly strategy for ion mass transfer applications.

## 2 Result and Discussion

### 2.1 Co‐Assembly of Three‐Component Small Molecules

**Figure 1:**
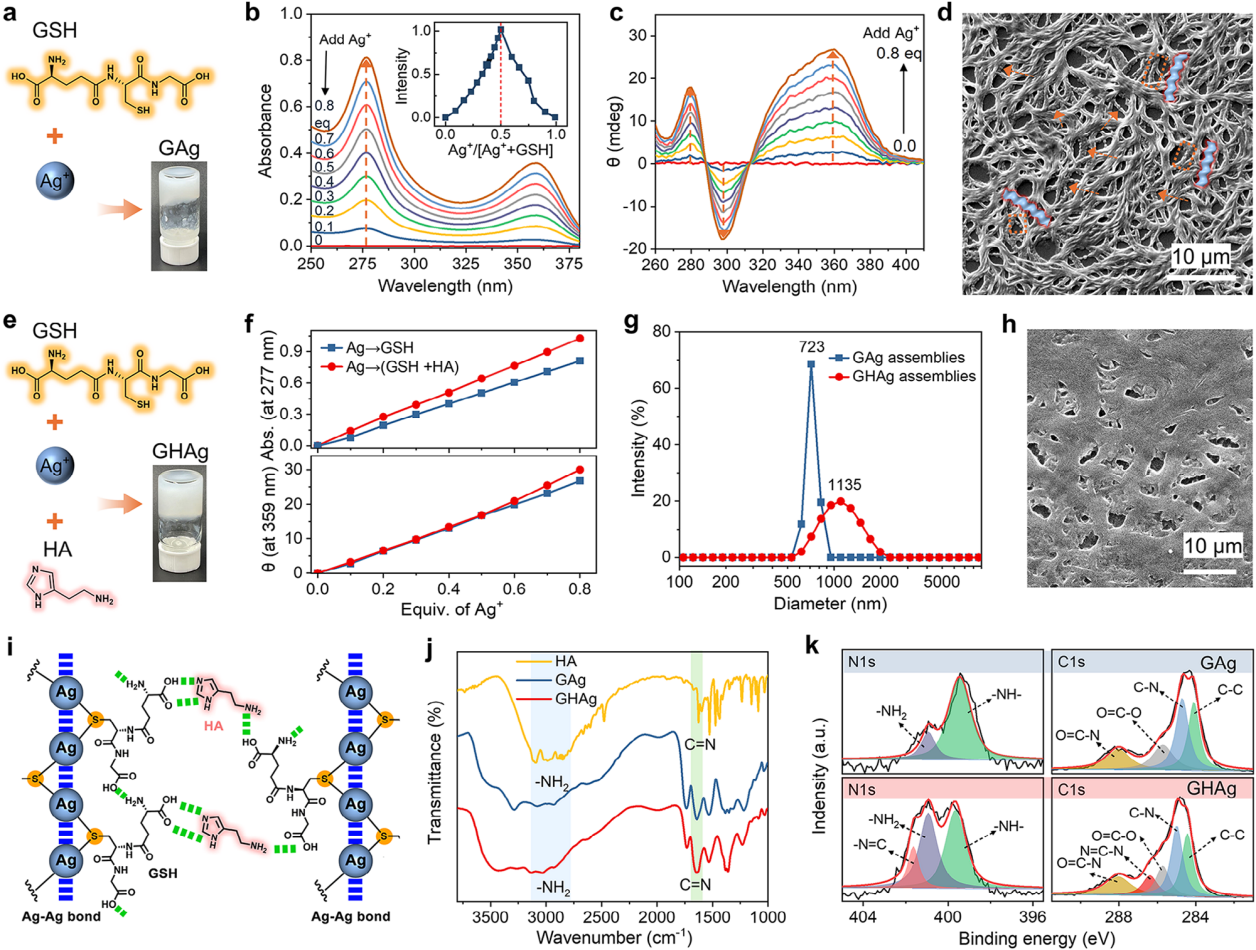
Co‐assembly of three‐component small molecules. a) Chemical structure of GSH, Ag^+^ ions, and a photograph showing the gel formed by binary co‐assembly. b) UV–vis and c) CD spectra of GSH solution after adding different equivalents of Ag^+^ ions. Inset: absorption intensity as a function of the relative molar content of Ag^+^ ions. d) SEM image of the GAg assemblies. e) Chemical structure of GSH and HA, Ag^+^ ions, and a photograph showing the gel formed by ternary co‐assembly. f) Changes in absorption intensity at 277 nm (UV–vis spectra, top panel) and in θ at 359 nm (CD spectra, bottom panel) for binary versus ternary systems. g) Size distribution of the two solid assemblies. h) SEM image of the GHAg assemblies. i) Schematic illustration of the possible coordination and complexation among GSH, Ag^+^ ions, and HA. Ag‐Ag bonds and hydrogen bonds are labeled with blue and green dashed lines, respectively. j) IR spectra and k) high‐resolution XPS spectra of the GAg and GHAg assemblies.

A nanofluidic membrane necessitates pervasive charges within its nanoconfined spaces to enable selective counter‐ions transport. To this end, we explored the co‐assembly of GSH and AgNO_3_ (**Figure** **1**a), since the thiol‐containing compounds are prone to coordinate with Ag^+^ ions to form co‐assembled nanowires,^[^ ^39^ ^]^ while Ag^+^ ions can bring abundant charges to the assemblies. UV–vis spectroscopy was first used to study the co‐assembly behavior by adding AgNO_3_ to the GSH solution (0.1 mm). As shown in Figure 1b, two absorption peaks at 277 and 359 nm gradually intensified with the continuous addition of AgNO_3_, which can be assigned to the ligand‐to‐metal charge–transfer transition.^[^ ^39^ ^]^ In contrast, no absorption peak was observed in the wavelength range of 250 to 450 nm for either GSH or AgNO_3_ alone. This indicates the formation of complexation or coordination between GSH and Ag^+^ ions. The Job plot of the absorption intensity at 277 nm with an inflection point at 50% suggests a 1:1 interaction stoichiometry between GSH and Ag^+^ ions, implying the formation of either a 1:1 complex or a (1:1)^n^ coordination polymer. Circular dichroism (CD) titration further confirmed the co‐assembly behavior (Figure 1c). Sequential addition of AgNO_3_ to the GSH solution generated two positive Cotton effects (276 and 350 nm) and one negative Cotton effect (295 nm), with all three peaks showing monotonic intensity enhancement throughout the titration. This observation indicates that helical chirality forms during the co‐assembly process. Then, scanning electron microscopy (SEM) was employed to observe the solid co‐assemblies. As displayed in Figure 1d, the co‐assembly formed thick, twisted helical nanofiber bundles. These findings, along with the CD experimental results, indicate that the point chirality of the GSH molecule is transferred to the helical chirality of the assemblies. Furthermore, when the concentration of each component in the mixture reaches 15 mm, the co‐assembly of GSH with Ag^+^ ions can form a visible gel after 12 h of incubation (Figure 1a, bottom right). Based on these results and the reported case,^[^ ^40^ ^]^ we presume that Ag^+^ ions coordinate with GSH by forming Ag^+^‐S bond, resulting in the formation of a coordination polymer capable of assembling into helical nanofibers.

When histamine (HA) was introduced into the assembly system (Figure 1e), we observed a significant enhancement in the assembly. As AgNO_3_ was continuously added to the mixed solution of GSH and HA (0.1 mm), the resulting UV–vis and CD spectra were comparable to those obtained by adding AgNO_3_ to the GSH solution (Figure S1, Supporting Information), indicating that the ternary system of GSH, Ag^+^ ions, and HA exhibits assembly behavior similar to that of the binary system. Notably, the rates of increase in the absorption intensity at 277 nm of UV–vis spectra (Figure 1f, top) and the θ at 359 nm of CD spectra (Figure 1f, bottom) of the ternary system are greater than those of the binary system, suggesting a stronger assembly capability of the ternary system. Then, dynamic light scattering (DLS) was employed to investigate the resulting assemblies in solution. The results suggest that the mean hydrodynamic size of the [GSH+Ag] (GAg, for short) assemblies is ≈723 nm (Figure 1g). In contrast, the [GSH+Ag+HA] (GHAg) assemblies give a markedly larger mean size (≈1135 nm), indicating that the inclusion of HA promotes co‐assembly. In addition, SEM observation shows that there is severe cross‐linking and denser assembly (Figure 1h), further confirming the co‐assembly of the ternary system. When the concentration of each component was increased to 15 mm, the ternary system also formed a gel (Figure 1e, bottom right).

Based on the above results, we propose a possible mechanism of the ternary co‐assembly. The coordination of GSH and Ag^+^ ions produced helical nanofibers, which were then entangled and cross‐linked by HA through multiple hydrogen bonds and/or electrostatic interactions between the HA imidazole and amine groups and GSH carboxyl groups, facilitating the rapid formation of larger and longer assemblies (Figure 1i). The solid assemblies formed in aqueous solutions were collected, dried, and characterized by infrared (IR) spectroscopy and X‐ray photoelectron spectroscopy (XPS) (Figure S2, Supporting Information). IR spectrum of the GHAg assemblies exhibits two distinct bands at ≈3007 cm^−1^ and 1628 cm^−1^ that correspond to the stretching vibrations of the –N–H and –C═N– bonds of HA, respectively (Figure 1j). Furthermore, the appearance of the N element from ─N═C─ and the increase of the N element from –NH_2_ in the N1s core‐level spectrum, as well as the appearance of the C elements from ─N═C─N– in the C1s core‐level spectrum were observed for the GHAg assemblies compared to GAg (Figure 1k). These results further confirm the engagement of HA in the ternary co‐assembly.

### 2.2 Preparation and Characterization of Heterogeneous Membranes

**Figure 2:**
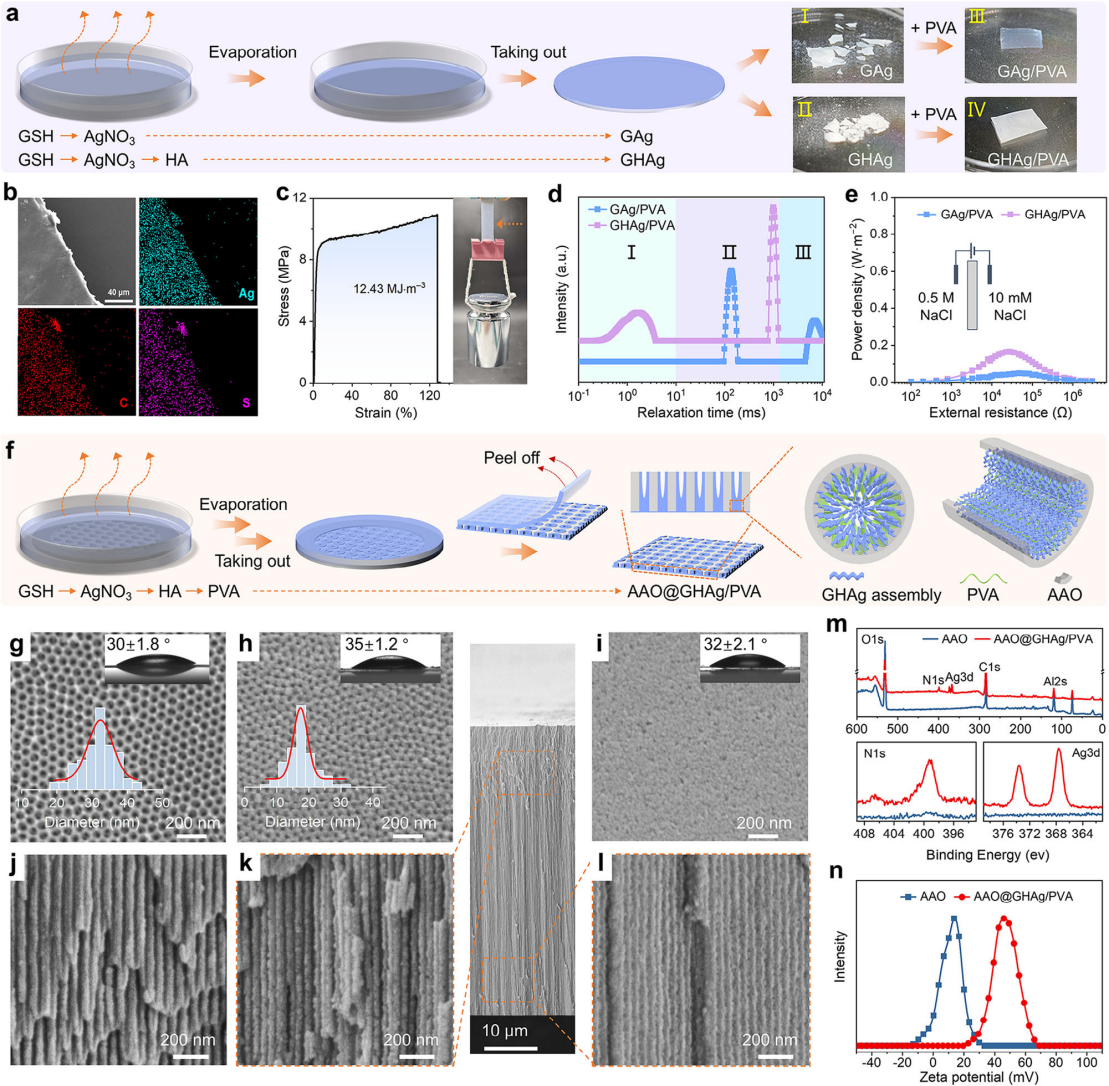
Fabrication of the heterogeneous membranes. a) Schematic illustration of the gel membrane preparation from direct co‐assembly or PVA addition, along with the membrane stability demonstrations (right photographs). b) SEM image and EDX elemental mappings of the GHAg/PVA membrane. c) The stress‐strain curve of the GHAg/PVA membrane and the photograph (right photograph) showing the weight‐lifting experiment. d) Low‐field NMR spectra of GAg/PVA and GHAg/PVA. I, II, and III represent sub‐nanoconfined (bound water), nanoconfined (immobilized water), and free space (free water), respectively. e) Output power density as a function of external resistance for the two gel membranes. f) Schematic illustration of the preparation of the heterogeneous membrane. g–l) SEM images of the surface of AAO (g), the top (h) and bottom (i) surface of AAO@GHAg/PVA, and the cross‐section of AAO (j), the upper (k) and lower (l) cross‐sections of AAO@GHAg/PVA. The lower insets of (g,h) are the channel size distributions. The upper insets of (g–i) are the surface water contact angle. m) XPS spectra and n) the zeta potential of AAO and AAO@GHAg/PVA. The zeta potential was measured by grinding the membrane into a fine powder and dispersing it in water.

Given that hydrogels can facilitate mass transfer (e.g., ions and molecules) through their internally interconnected nanochannels,^[^ ^41^, ^42^, ^43^ ^]^ we first explored the direct construction of the gel membranes from the above co‐assembly systems. Specifically, a mixture solution containing equal concentrations (15 mm) of each component was prepared and then poured into a Petri dish (60 mm diameter × 15 mm depth) and left for three days to form a gel membrane (**Figure** **2**a). However, both the resulting binary GAg and the ternary GHAg membranes were unstable and disintegrated after overnight immersion in water (Figure 2a, I and II). We then attempted to improve their stability by introducing PVA (0.5 wt.%) into the co‐assembly process because of its exceptional ability to colligate nanorods or nanofibers by entangling them.^[^ ^44^ ^]^ We found that the resulting gel membranes, namely the GAg/PVA and GHAg/PVA membranes, exhibit excellent water stability (Figure 2a, III and IV) and still maintain high hydrophilicity (Figure S3, Supporting Information). Energy dispersive X‐ray (EDX) elemental mappings of the GHAg/PVA (Figure 2b) and GAg/PVA (Figure S4, Supporting Information) gel membranes clearly show a uniform dispersion of Ag, C, and S elements. Importantly, the incorporation of PVA dramatically improved the mechanical strength. For example, a tensile strength of 10.9 MPa, a strain‐to‐failure of 128%, and a toughness of 12.43 MJ·m^−3^ were measured for the GHAg/PVA membrane (Figure 2c).

Low‐field nuclear magnetic resonance (NMR) was used to study the gel membranes. This technique measures the transverse relaxation time (*T*_2_) of water molecules to provide information about the water status within the materials.^[^ ^45^ ^]^ As shown in Figure 2d, *T*_2_ for the GAg/PVA membrane shows two sharp peaks centered at 105 and 7,000 ms, respectively. The former represents immobilized water in the nanoconfined space, while the latter represents bulk water in the free space (i.e., free water). In contrast, no peak corresponding to the free water was observed for the GHAg/PVA membrane, but a broad peak belonging to bound water was found at a short relaxation time (≈1.5 ms). This indicates more hydrophilic groups in the GHAg/PVA membrane due to the introduction of HA. In addition, GHAg/PVA shows a sharp peak at ≈1,000 ms, corresponding to immobilized water. This T_2_ value was much longer than that of GAg/PVA, suggesting larger nanoconfined spaces for water in GHAg/PVA.^[^ ^46^ ^]^ We then evaluated the osmotic energy harvesting capacity of the gel membranes by measuring the current‒voltage (*I*‒*V*) curves in the presence of an external resistance (Figure S5, Supporting Information).^[^ ^37^ ^]^ However, to our disappointment, the output power of either the GAg/PVA or GHAg/PVA membranes in a simulated seawater (0.5 M NaCl)/river water (10 mM NaCl) system was very low, with the peak values of only 0.05 and 0.17 W·m^−2^, respectively (Figure 2e). This might be attributed to the densely stacked structure of the gel membranes, significantly hampering the ion transport.^[^ ^47^ ^]^

Nanoporous AAO membranes provide an excellent platform for constructing various types of nanofluidic membranes.^[^ ^48^, ^49^, ^50^ ^]^ With this in mind, we next attempted to perform in situ co‐assembly within AAO channels to prepare a heterogeneous membrane. Figure 2f illustrates the preparation procedure (see details in Supporting Information). First, a precursor solution was prepared by sequentially mixing stock solutions of GSH, AgNO_3_, HA, and PVA. The solution was then poured into a Petri dish containing a clean and hydrophilic AAO membrane (channel diameter: 30−40 nm; mean: 32 nm, thickness: 60 µm) at the bottom. Gentle shaking facilitated penetration into the AAO channels. Note that the AAO membrane was pre‐fixed to the bottom of the Petri dish using sticky tape (Figure S6, Supporting Information), ensuring that the solution only came into contact with the top side of AAO, while the bottom side remained undisturbed. After three days of assembly and solvent evaporation, the AAO membrane was taken out. After peeling off the gel‐like layer covering the AAO top surface and rinsing with water, the heterogeneous membrane, AAO@GHAg/PVA, was obtained. The formed assemblies were firmly anchored in the AAO channels by PVA's adhesive properties, along with potential hydrogen bonding, coordination interactions, and/or van der Waals forces between the assemblies and channel walls.

The pristine AAO channels have a mean diameter of ≈32 nm (Figure 2g,j). After loading the GHAg/PVA assemblies, the mean channel size of the top surface decreased to ≈18 nm (Figure 2h), in stark contrast to the almost completely blocked bottom surface channels (Figure 2i). Additionally, cross‐sectional observations revealed wider upper channels compared to the lower ones (Figure 2k,l). Complementing this observation, EDX analysis revealed substantially greater Ag element accumulation in the lower part of the channel compared to the upper part (Figure S7, Supporting Information). Together, the channel size disparity and differential Ag distribution provide further evidence of the asymmetric channel structure. XPS spectrum of AAO@GHAg/PVA shows clear peaks belonging to N1s and Ag3d, supporting successful loading of the co‐assemblies (Figure 2m). The N_2_ sorption isotherms display a type‐IV behavior, indicating the ordered mesoporous structure of the AAO@GHAg/PVA membrane (Figure S8a, Supporting Information). The Brunauer‐Emmett‐Teller (BET) surface area was calculated to be 25.7 m^2^·g^−1^. A peak value of 1.4 nm was observed in the pore distribution curve, confirming that the membrane possesses subnanoscale channels (Figure S8b, Supporting Information).^[^ ^9^ ^]^ The zeta potential of AAO@GHAg/PVA in a neutral aqueous solution is approximately +46.0 mV (Figure 2n), indicating its positively charged property.

### 2.3 Ion Transport Behaviors of the Heterogeneous Membrane

**Figure 3:**
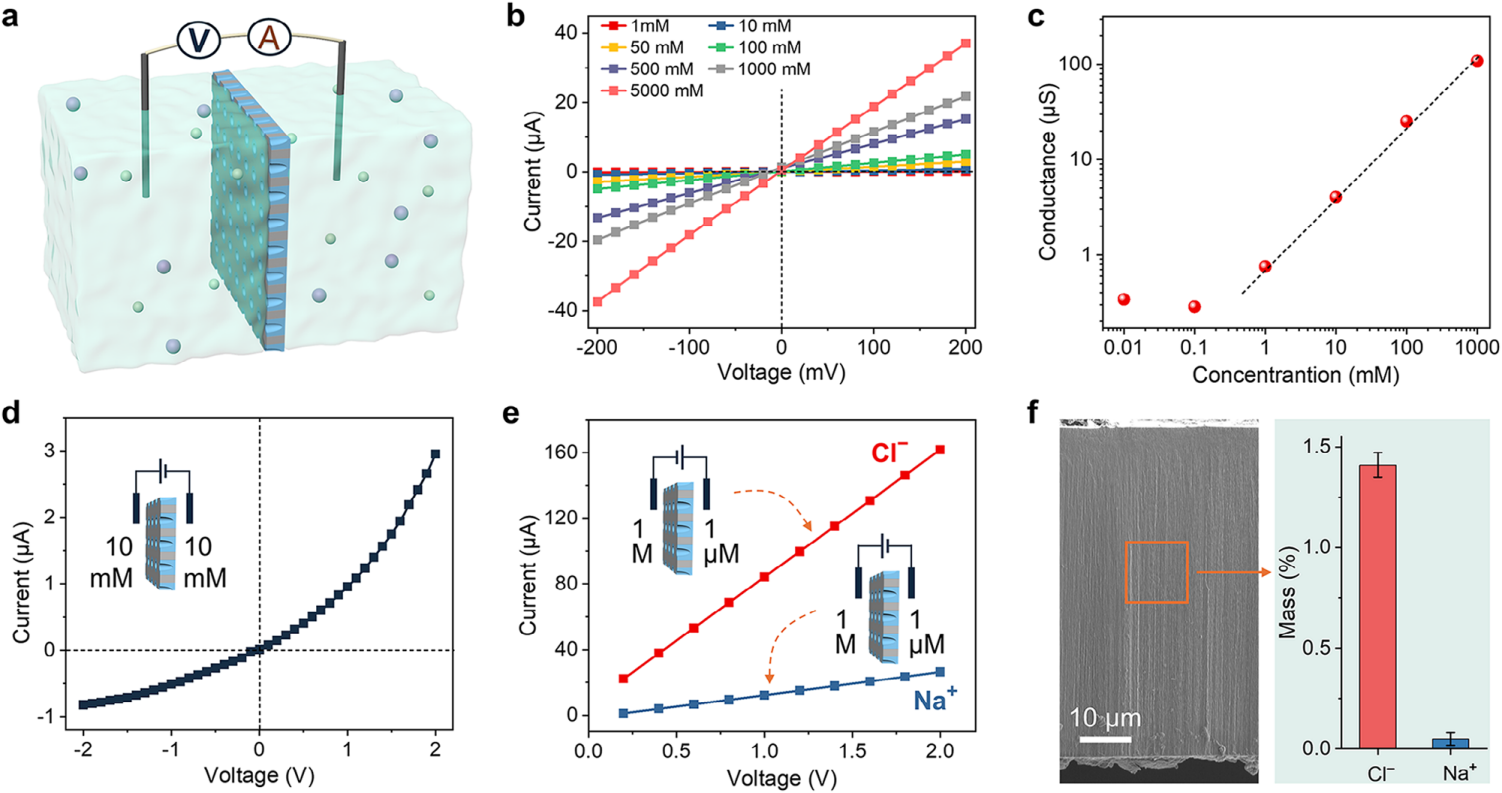
Ion transport properties of heterogeneous membrane. a) Schematic illustration of the custom‐made electrochemical test device. b) *I*–*V* curves and c) ionic conductance data of AAO@GHAg/PVA under different NaCl concentrations. d) *I*–*V* curve of AAO@GHAg/PVA measured in symmetric 10 mm NaCl solutions over a voltage range from –2 to +2 V. e) Two *I*–*V* curves of AAO@GHAg/PVA under a 1 M/1 µM NaCl gradient over a voltage range from +0.2 to +2 V recorded by reversing the electrodes. f) The relative content of Cl^−^ and Na^+^ in the heterogeneous membrane from EDX elemental mapping of the cross‐section of AAO@GHAg/PVA after overnight immersion in a 1 m NaCl solution. Error bars in panel (f–i) represent standard deviations (n=3).

Ion transport property of the AAO@GHAg/PVA heterogeneous membrane was investigated using a custom‐made electrochemical device (**Figure** **3**a). Figure 3b shows the recorded *I*‒*V* curves of AAO@GHAg/PVA at different NaCl concentrations. The ionic conductance decreases nonlinearly with decreasing salt concentration (Figure 3c), especially when the concentration is below 1 mm, suggesting that AAO@GHAg/PVA exhibits charge‐controlled ion transport behavior at low salt concentrations. Furthermore, an asymmetric *I*‒*V* curve with a rectification ratio of ≈3.6 was observed in symmetric 10 mm NaCl solutions over a wide voltage range from ‒2 V to +2 V (Figure 3d). The observed asymmetric *I*‒*V* characteristic represents a hallmark of diode‐like ionic current rectification (ICR), reflecting asymmetric ion transport behavior across the AAO@GHAg/PVA membrane. Given that ICR effects in nanofluidic systems generally stem from geometric or charge distribution asymmetries,^[^ ^51^ ^]^ this finding further confirms the asymmetric nanochannel structure of AAO@GHAg/PVA. The ICR effect is expected to facilitate the selective, directional, and amplified ion transport while weakening the ion polarization phenomenon, thereby potentially improving power generation in osmotic energy conversion.^[^ ^51^, ^52^ ^]^

We then measured *I*‒*V* curves using a 1 m NaCl solution on the large‐opening side of AAO@GHAg/PVA and 1 µm NaCl solution on the blocked‐channel side. Under this exceptionally large concentration gradient, the ionic current is predominantly generated by the ion flux from the high to low concentration side.^[^ ^9^ ^]^ Figure 3e shows two *I*‒*V* curves recorded over the voltage range of +0.2 to +2 V by reversing electrodes. The Cl^−^ ion‐generated current is significantly higher than that of Na^+^ ions, confirming the anion selectivity of AAO@GHAg/PVA. In addition, a freshly prepared AAO@GHAg/PVA membrane was subjected to EDX characterization of its cross‐section before and after being immersed in a 1 M NaCl solution overnight. The significantly higher content of Cl^−^ ions compared to Na^+^ ions within the membrane further confirms the anion selectivity (Figure 3f; Figure S9, Supporting Information).

### 2.4 Osmotic Energy Harvesting Performance Evaluation

**Figure 4:**
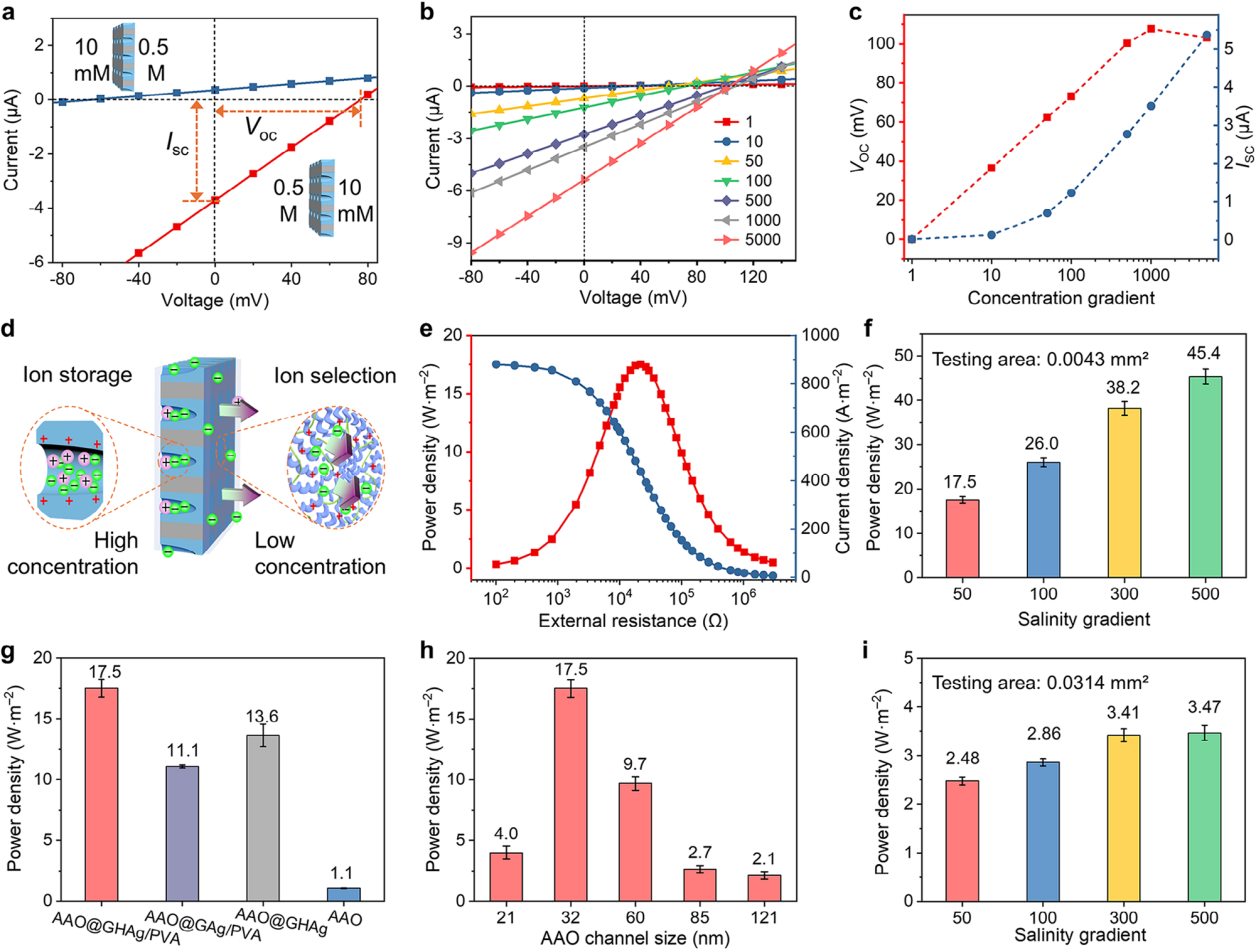
Osmotic energy harvesting performance of AAO@GHAg/PVA. a) *I*–*V* curves of AAO@GHAg/PVA under forward and reverse 50‐fold NaCl gradients. b) *I*–*V* curves and c) *V*_oc_ and *I*_sc_ values under different NaCl gradients. d) Schematic illustrating the proposed selective ion transport mechanisms across the AAO@GHAg/PVA membrane. e) Output current and power densities as functions of external resistance for AAO@GHAg/PVA under a 50‐fold NaCl gradient. f) Peak power densities of AAO@GHAg/PVA under different NaCl gradients (effective testing area: 0.0043 mm^2^). g) Peak power densities of AAO@GHAg/PVA, AAO@GAg/PVA, AAO@GHAg, and AAO under a 50‐fold NaCl gradient. h) Peak power densities of AAO@GHAg/PVA fabricated from AAO substrates with varying channel diameters under a 50‐fold NaCl gradient. i) Peak power densities of AAO@GHAg/PVA under different NaCl gradients (effective testing area: 0.0314 mm^2^). Error bars in panels (f–i) represent standard deviations (n=3).

Next, we evaluated the osmotic energy conversion of AAO@GHAg/PVA by measuring *I*‒*V* curves with a concentration gradient of NaCl across the membrane. **Figure** **4**a shows two *I*‒*V* curves of AAO@GHAg/PVA obtained by reversing the concentration gradient (0.5 m versus 10 mM, i.e., 50‐fold salinity gradient). The asymmetry of the two *I*‒*V* curves further demonstrates the structural asymmetry of AAO@GHAg/PVA nanochannels, which echoes the ICR characteristics shown in Figure 3d. When the 0.5 m NaCl solution was placed on the large‐opening side of AAO@GHAg/PVA, the significantly higher open‐circuit voltage (*V*_oc_) and short‐circuit current (*I*_sc_) values were generated. Of particular note, a pair of Ag/AgCl electrodes containing the saturated salt solutions as a salt bridge was used in order to eliminate the potential created by redox reactions on the electrodes under asymmetric salinity conditions.^[^ ^17^, ^53^ ^]^ As a result, the obtained *V*_oc_ and *I*_sc_ values correspond directly to the osmotic potential and current, respectively.^[^ ^53^ ^]^ Comparative analysis of the *I‒V* curves reveals that AAO@GHAg/PVA achieves significantly higher *V*_oc_ and *I*_sc_ values than the gel membrane (GHAg/PVA) without the AAO substrate (Figure S10, Supporting Information). This indicates that the introduction of AAO substrate significantly improves both ion selectivity and ion permeability of the heterogeneous membrane.^[^ ^54^ ^]^ In addition, we compared the *I‒V* characteristics of AAO@GHAg/PVA and the pristine AAO membrane. The *V*_oc_ and *I*_sc_ values of AAO@GHAg/PVA are also significantly higher than those of AAO (Figure S11a, Supporting Information). The enhanced *V*_oc_ can be attributed to the increase of ion selectivity due to the introduction of the ternary assemblies with enriched positive charges. Intuitively, the smaller *I*_sc_ of AAO indicates its poorer ion permeability. However, the ion conductance measurements showed that AAO has a significantly higher ion conductance (≈35 µS at 0.1 m NaCl) than AAO@GHAg/PVA (≈25 µS at 0.1 m NaCl) (Figure S11b, Supporting Information). This aligns with expectations, as larger channels/pores of AAO facilitate easier ion transport. Thus, the smaller *I*_sc_ of AAO should be attributed to its poor ion selectivity, which allows the simultaneous transport of positive and negative ions from high to low concentration, leading to a smaller net osmotic current.^[^ ^52^, ^55^ ^]^ These findings further confirm that the ternary co‐assemblies anchored in the AAO channels endows the AAO@GHAg/PVA membrane with excellent ion permselectivity. In subsequent experiments, the high‐concentration saline solution was applied to the large‐opening side of AAO@GHAg/PVA to evaluate its performance.

The *I‒V* curves were measured under a series of salinity gradients (Figure 4b), with the high concentration ranging from 0.001, 0.01, 0.05, 0.1, 0.5, 1 to 5 m NaCl and the low concentration fixed at 1 mm NaCl (i.e., from 1‐ to 5000‐fold NaCl gradient). Figure 4c shows that both *V*_oc_ and *I*_sc_ increased with the salinity gradient, with the exception of a slight drop in *V*_oc_ at 5000‐fold gradient. The anion transference number (*t*_−_) varied from 0.814 to 0.803 for salinity gradients ranging from 10‐ to 1000‐fold gradient (Table S1, Supporting Information). The corresponding energy conversion efficiency (*η*) ranged between 18.4% and 19.7%, but dropped sharply to 11.1% at the 5000‐fold gradient. This reduction occurs mainly because the surface charge is shielded in a highly concentrated solution, which decreases the electrical double layer thickness and degrades anion selectivity (Table S2, Supporting Information).^[^ ^56^ ^]^ Furthermore, the asymmetric nanochannel structure of the AAO@GHAg/PVA membrane plays a critical role in facilitating efficient ion transport, as documented in a previous study.^[^ ^8^ ^]^ The large‐opening side—exposed to the high‐concentration solution—contains residual long nanochannels that serve as reservoirs for a large number of ions (Figure 4d). The positive charge property of the channel wall prefers negative ions. The narrower small‐opening side acts as an ion screen, selectively allowing negative ions to pass. Consequently, the asymmetric nanochannel structure of AAO@GHAg/PVA facilitates its osmotic energy harvesting.

To evaluate the osmotic energy harvesting performance in detail, an adjustable external resistance (*R*_L_) was introduced as a load in a circuit containing an electrochemical device equipped with an AAO@GHAg/PVA membrane (Figure S12, Supporting Information). The output power density (*P*) can be calculated using the equation *P* = *I* ^2^ × *R*_L_ / *S*_m_, where *I* is the measured current and *S*_m_ represents the effective membrane testing area. Under a 50‐fold NaCl gradient, the output power density reached its peak value of 17.5 W·m^−2^ when the load resistance was ≈20.9 kΩ (Figure 4e). In addition, the power density increased with salinity gradient, reaching up to 45.4 W·m^−2^ under a 500‐fold gradient (Figure 4f; Figure S13, Supporting Information). However, under the same condition, the AAO@GAg/PVA membrane from the binary co‐assembly generated a peak power density of ≈11.1 W·m^−2^ (Figure 4g; Figure S14, Supporting Information). In sharp contrast, the AAO produced a peak power density of only 1.1 W·m^−2^ (Figure S15, Supporting Information). These results further confirm the important role of co‐assemblies loaded in AAO nanochannels. Additionally, a PVA‐free heterogeneous membrane (AAO@GHAg) was also prepared, which initially generated a peak power density of 13.6 W·m^−2^ (Figure 4g). However, the lack of PVA resulted in rapid performance deterioration, retaining only 4.2 W·m^−2^ after 4 h (Figure S16, Supporting Information). This result underscores the critical role of PVA in maintaining membrane stability.

Next, the influence of the AAO channel size on membrane performance was examined. A series of AAO@GHAg/PVA membranes were prepared using AAO substrates with mean channel diameters of ≈21, 32, 60, 85, and 121 nm (Figure S17, Supporting Information). The test results show that AAO@GHAg/PVA exhibits the highest power density at an AAO substrate with a mean channel size of 32 nm (Figure 4h; Figure S18, Supporting Information). When the channel size increased, the power density decreased gradually. In larger channels, the assembled structure can grow denser, similar to the bulk gel membrane with a compact stacking structure, thereby hindering ion transport. When the AAO channel size reduced to 21 nm, the power density decreased compared to the 32‐nm‐diameter membrane. We presume that smaller AAO channels influence the infiltration of the precursor solution, leading to incomplete pore filling, thereby impairing ion permselectivity. Furthermore, the effect of the AAO thickness was also investigated. A 40‐µm‐thick AAO substrate (channel diameter: 30‐40 nm) was used to prepare the AAO@GHAg/PVA membrane. The resulting membrane achieved a higher power density of 21.0 W·m^−2^ under a 50‐fold NaCl gradient (Figure S19, Supporting Information), exceeding that of the 60‐µm‐thick substrate with comparable channel sizes. Thinner membranes shorten ion transport paths and improve the ion permeability, thereby enhancing power output. However, although the thinner membrane demonstrates improved performance, the reduced mechanical durability due to increased fragility presents practical limitations. Therefore, all subsequent experiments employed 60‐µm‐thick AAO substrates with a mean channel size of 32 nm to fabricate heterogeneous membranes.

The above output power densities were measured using an effective test area of 0.0043 mm^2^. We then adopted a larger effective test area of ≈0.0314 mm^2^. The heterogeneous membrane exhibits a peak power density of 2.48 W·m^−2^ under a 50‐fold NaCl gradient and 3.47 W·m^−2^ under a 500‐fold gradient (Figure 4i; Figure S20, Supporting Information). With an even larger test area (1.13 mm^2^), the power density further dropped to 0.67 W·m^−2^ (Figure S21a, Supporting Information). As the test membrane area increases, the output power density decreases, even though the total output power rises (Figure S21b, Supporting Information). This negative correlation between power density and membrane area primarily arises from intensified concentration polarization at larger scales, which reduces ion permselectivity and consequently diminishes power output.^[^ ^57^ ^]^

### 2.5 Assessing Ion Selectivity, Anti‐Biofouling Activity, and Stability

**Figure 5:**
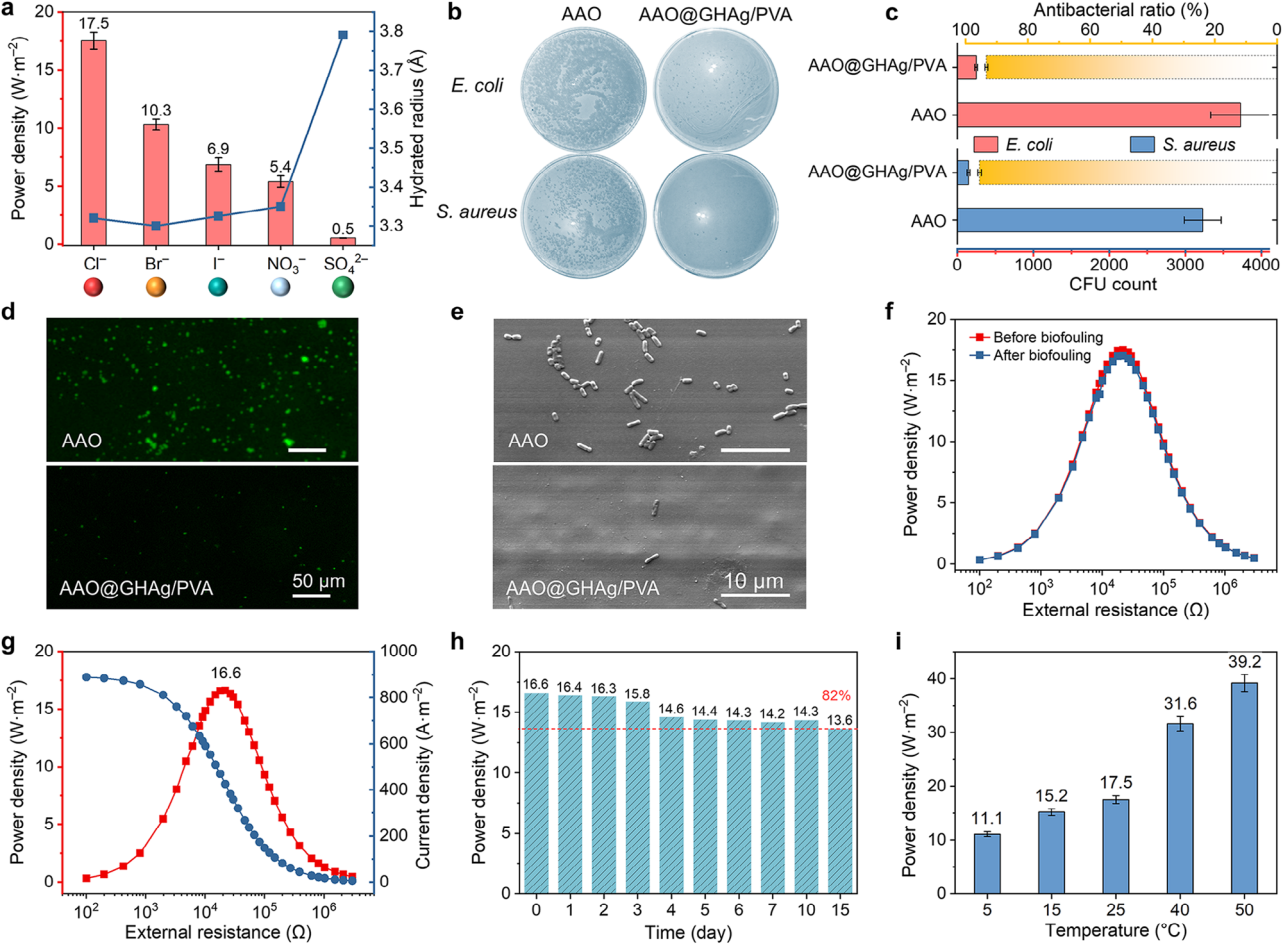
Ion selectivity, anti‐biofouling activity, and stability. a) Peak power densities of AAO@GHAg/PVA under a 50‐fold salinity gradient toward different anions. b) Photographs of *E. coli* and *S. aureus* colonies after 10 h culture following treatment with AAO or AAO@GHAg/PVA membranes. c) Antibacterial efficiency of the AAO and AAO@GHAg/PVA membranes. d) Fluorescence and e) SEM images of *E. coli* (green) attached to the AAO and AAO@GHAg/PVA membranes. f) Output power densities of AAO@GHAg/PVA tested before and after biofouling (bacterial attachment) under a 50‐fold NaCl gradient. g) Output current and power of AAO@GHAg/PVA in a natural seawater/river water system, h) and peak power densities recorded during 15 days of testing. i) Peak power densities of AAO@GHAg/PVA under a 50‐fold NaCl gradient at different temperatures. Error bars in panels (a,c,i) represent standard deviations (n=3).

Given the diverse ion composition of seawater, we next evaluated the performance of the AAO@GHAg/PVA membrane toward different anions. Sodium salts with different anions, i.e., NaCl, NaBr, NaI, NaNO_3_, and Na_2_SO_4_, were used to prepare salt solutions. The output current and power of AAO@GHAg/PVA were measured under a constant 50‐fold salinity gradient (Figure S22, Supporting Information). The produced peak output power densities are 17.5, 10.3, 6.9, 5.4, and 0.5 W·m^−2^, for Cl^−^, Br^−^, I^−^, NO_3_^−^, and SO_4_^2−^, respectively (**Figure** **5**a). To evaluate membrane selectivity, we calculated the power ratio by comparing each salt's performance against the Na_2_SO_4_ baseline [i.e., *P*(salt) / *P*(Na_2_SO_4_)], since the membrane demonstrated minimal power generation with sulfate electrolytes.^[^ ^58^ ^]^ The resulting selectivity ratios were determined to be 35.0 (Cl^−^/SO_4_^2−^), 20.6 (Br^−^/SO_4_^2−^), 13.8 (I^−^/SO_4_^2−^), and 10.8 (NO_3_^−^/SO_4_^2−^). All ions in aqueous solutions are known to be hydrated. The hydration radii for the relevant anions are as follows: Cl^−^ (3.32 Å), Br^−^ (3.30 Å), I^−^ (3.32 Å), NO_3_^−^ (3.35 Å), and SO_4_^2−^ (3.79 Å). It can be roughly observed that the larger the hydration radius of the ions, the lower the output power. Overall, the anion with a smaller hydration radius can transport across the membrane more easily, leading to more efficient anion permeability and higher power output.^[^ ^59^ ^]^ Besides hydrated radius, the osmotic power conversion performance toward various anions is also collectively influenced by multiple factors, such as dynamic ion‐membrane interactions and ionic diffusivity.^[^ ^58^ ^]^

Since natural water contains a variety of microorganisms, anti‐biofouling activity is one of the essential skills of nanofluidic membranes.^[^ ^60^ ^]^ The first and most critical stage in the biofouling process is the settling and growth of bacteria on the material surface to form a biofilm.^[^ ^61^ ^]^ Preventing bacterial attachment or even killing bacteria therefore becomes the key to material resistance to biofouling. Given silver's well‐documented broad‐spectrum antimicrobial properties, we evaluated the antibacterial performance of AAO@GHAg/PVA using Gram‐negative *E. coli* and Gram‐positive *S. aureus* as model organisms. Membranes were co‐cultivated with the bacterial strains for 12 h and then the bacterial concentration attached to the membrane surface was determined by the dilution plate counting method after collection and re‐cultivation. Results demonstrate significantly fewer bacterial colonies for both strains with AAO@GHAg/PVA compared to pristine AAO (Figure 5b). Colony forming unit (CFU) analysis revealed 94.0% antibacterial efficiency against *E. coli* and 95.5% against *S. aureus* relative to AAO controls (Figure 5c). Furthermore, fluorescence and SEM images of membranes after 12 h co‐cultivation and PBS rinsing showed minimal bacterial retention on AAO@GHAg/PVA versus AAO (Figure 5d,e), confirming superior antibacterial activity. Performance testing before/after 12 h co‐cultivation with *E. coli* demonstrated only 2.8% power reduction and stable internal resistance (Figure 5f), validating the superior biofouling resistance of AAO@GHAg/PVA and its potential for real‐world applications.

Next, we assessed the performance of AAO@GHAg/PVA in natural seawater/river water systems (Figure 5g). The membrane reached a peak power density of 16.6 W·m^−2^ and maintained 82% of its initial performance over 15 days (Figure 5h), demonstrating stability. Additionally, the effect of temperature on performance was evaluated (see Supporting Information for testing details). As shown in Figure 5i, the output power increased with temperature and reached 39.2 W·m^−2^ at 50 °C, 2.2 times higher than at room temperature (Figure S23, Supporting Information). This enhancement can be attributed to the enhanced ion diffusion coefficient and the reduced fluid viscosity at high temperatures. The heterogeneous membrane also maintains thermal stability over the experimental temperature range (Figure S24, Supporting Information). The performance enhancement of the membrane at high temperatures makes it suitable for some specific scenarios, for example, hydrogen production via seawater electrolysis^[^ ^62^ ^]^ and seawater desalination,^[^ ^63^ ^]^ as these processes produce a considerable amount of high‐temperature and high‐salinity effluent.

### 2.6 Extensibility of the Building Blocks of Ternary Co‐Assembly

**Figure 6:**
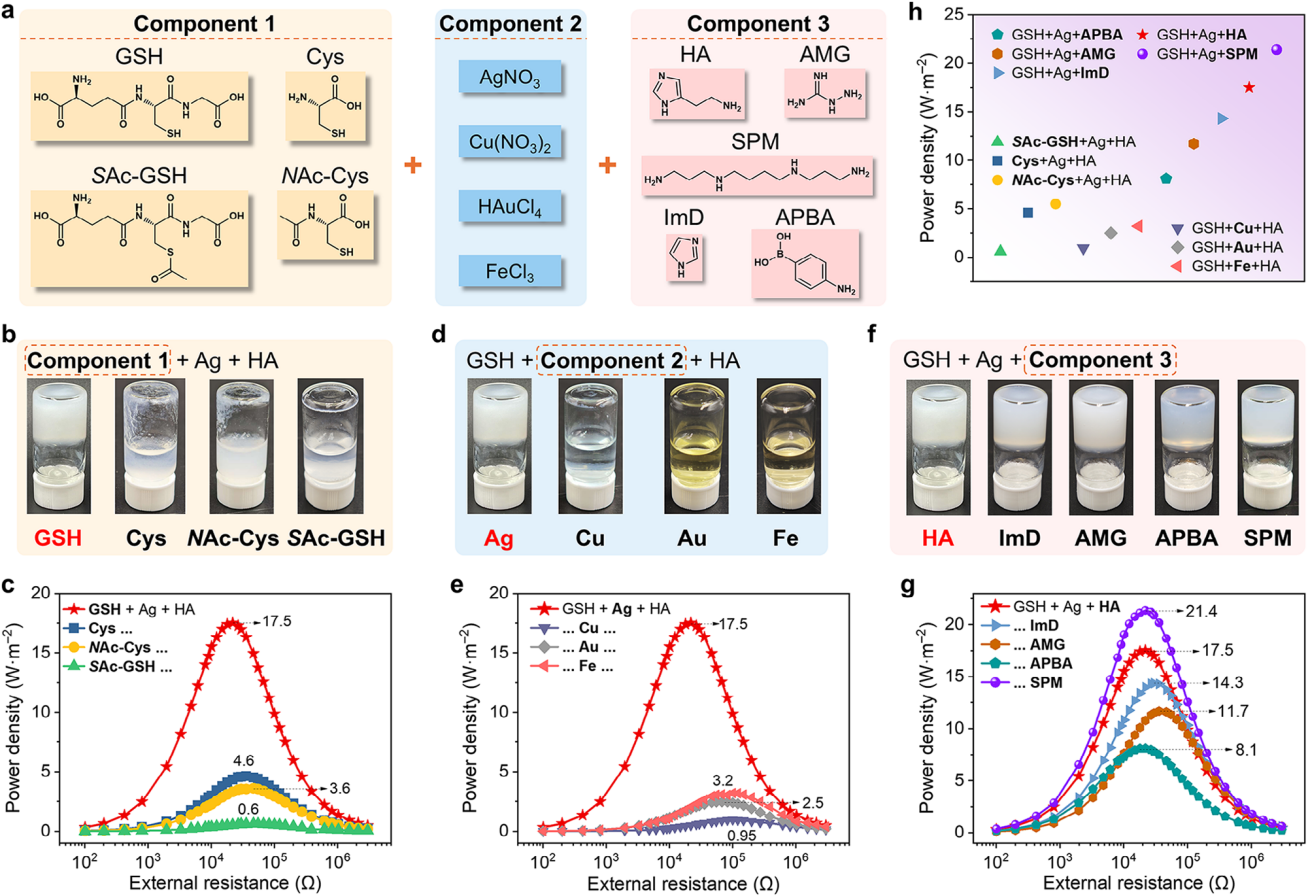
Extensibility of the building blocks. a) Chemical structure of different molecule precursors, and salts to provide ions. b,d,f) Photographs showing mixture solutions or gels by changing one component after one hour of incubation. The cloudy solutions are due to the poor solubility of *S*Ac‐GSH and *N*Ac‐Cys at a high concentration (15 mM). c,e,g) External resistance‐dependent output power densities of different heterogeneous membranes from co‐assembly in AAO channels or deposition on AAO substrates under a 50‐fold NaCl gradient. h) Comparison of peak power densities among different membranes.

We next sought to extend the building blocks of the ternary co‐assembly system. First, GSH was replaced with various analogues—including L‐cysteine (Cys), *S*‐acetyl‐L‐glutathione (*S*Ac‐GSH), and *N*‐acetyl‐L‐cysteine (*N*Ac‐Cys)—to participate in co‐assembly (**Figure** **6**a, component 1). The UV–vis titration experiment revealed that Cys and Ag^+^ ions underwent a co‐assembly similar to that of GSH and Ag^+^ ions (Figure S25, Supporting Information). However, subsequent HA addition did not yield ternary co‐assembly (Figure S25f, Supporting Information). And the [Cys+Ag+HA] mixture failed to form a gel after one hour of incubation (Figure 6b). Then, the heterogeneous membrane preparation method (Figure 2f) was applied to the [Cys+Ag+HA] system. However, the resulting membrane exhibits a peak output power density of only 4.6 W·m^−2^ (Figure 6c), substantially lower than that of AAO@GHAg/PVA (derived from the [GSH+Ag+HA] co‐assembly). This finding indicates the rationality of ternary co‐assembly for constructing a high‐efficiency osmotic energy harvesting membrane. Additionally, UV–vis titrations confirmed that neither [*S*Ac‐GSH+Ag+HA] nor [*N*Ac‐Cys+Ag+HA] could effectively co‐assemble (Figure S26, Supporting Information). In the case of *S*Ac‐GSH, the thiol group is shielded, preventing S^−^‐Ag^+^ coordination. For *N*Ac‐Cys, the acetylated amine group failed to form hydrogen bonds with the neighboring carboxylic acid to shorten the distance between Ag atoms, impeding coordination formation. Gel formation tests further supported these findings, as both mixtures (15 mm) remained clear after one hour (Figure 6b). For comparison, the heterogeneous membranes of [*S*Ac‐GSH+Ag+HA] and [*N*Ac‐Cys+Ag+HA] were also prepared by depositing their mixture solution on AAO substrates and drying for three days. As expected, the peak power densities of [*S*Ac‐GSH+Ag+HA] and [*N*Ac‐Cys+Ag+HA] were only 0.6 and 3.6 W·m^−2^, respectively, confirming their poor energy conversion efficiency. These findings underscore the importance of GSH's functional groups in enabling efficient ternary co‐assembly, as well as the importance of assemblies for membrane performance.

Then Cu^2+^ (Cu(NO_3_)_2_), AuCl_4_^−^ (HAuCl_4_), and Fe^3+^ (FeCl_3_) (Figure 6a, component 2) were respectively used to replace the Ag^+^ ion (AgNO_3_) to participate in the co‐assembly with GSH and HA. None of these systems were able to form an assembly, as indicated by the clear mixed solutions and the almost unchanged UV–vis spectra (Figure 6d; Figure S27, Supporting Information). Under these circumstances, all of the resulting deposited membranes produced low output power (Figure 6e). Additionally, the mixed [GSH+HA] solution remained clear in the absence of Ag⁺, confirming no assembly formation. The resulting deposited membrane AAO@GH/PVA produced a maximum output power density of only 0.26 W·m^−2^ (Figure S28, Supporting Information). These results demonstrate that Ag⁺ plays an essential role in assembly formation, and the assemblies are crucial for the osmotic energy harvesting of the heterogeneous membrane.

Furthermore, attempts were made to replace the HA with other amino‐containing compounds, e.g., imidazole (ImD), aminoguanidine (AMG), 4‐aminophenylboronic acid (APBA), and spermine (SPM) (Figure 6a, component 3). We found that, similar to HA, the participation of ImD, AMG, APBA, or SPM also promoted the co‐assembly of GSH and Ag⁺ ions, as evidenced by the enhanced co‐assembly effects observed in the UV–vis spectra (Figure S29, Supporting Information). When the mixture solution contains higher concentrations of each component, these compounds also promoted gel formation (Figure 6f). The resulting heterogeneous membranes from [GSH+Ag+ImD] and [GSH+Ag+AMG] produced the peak power densities of 14.3 and 11.7 W·m^−2^, respectively (Figure 6g). In contrast, the maximum power density of the membranes from [GSH+Ag+APBA] is slightly inferior, only 8.1 W·m^−2^, which could be attributed to the negatively charged boronic acid groups that offset part of the anion selectivity. It is worth noting that the peak power density of the membrane from [GSH+Ag+SPM] is up to 21.4 W·m^−2^ (Figure 6g,h). Four amino groups (their pK_a_ ranges: 10.5–11.0, 9.5–10.0, 8.5–9.0, and 7.5–8.0) of SPM are fully protonated in aqueous solution (pH 6.5). This results in a higher positive charge density of the heterogeneous membrane, as confirmed by the zeta potential of +49.2 mV (Figure S30, Supporting Information). Additionally, the long and flexible chain of SPM might facilitate more crosslinking between the coordination assemblies of GSH and Ag^+^ ions, thus producing more ion‐selective transport channels and increasing ion permselectivity (Figure S31, Supporting Information).

## 3 Conclusion

In summary, we have demonstrated a heterogeneous membrane based on the ternary co‐assembly of GSH, HA, and Ag ions within AAO nanochannels along with the participation and reinforcement of PVA. The heterogeneous membrane features an asymmetric nanochannel structure, with one side remaining open while the opposite side is nearly blocked. This structural asymmetry, combined with the positively charged nature, facilitates diode‐like anion‐selective transport across the membrane. The membrane achieves excellent performance, with a maximum output power density of 17.5 W·m^−2^ in a simulated seawater/river water system, and also exhibits superior anti‐biofouling activity. Interestingly, the ternary co‐assembly system is extensible and adaptable by replacing or adding new molecule or ion precursors, allowing for further performance improvement.

In contrast to conventional nanofluidic membranes prepared via vacuum suction filtration^[^ ^8^, ^9^, ^11^, ^12^, ^13^, ^21^, ^22^, ^23^ ^]^ or complex chemical synthesis,^[^ ^14^, ^15^, ^16^, ^17^, ^18^, ^19^, ^24^, ^25^ ^]^ this work establishes a new fabrication paradigm through a molecular co‐assembly strategy. This approach offers distinct advantages, including flexible component design, facile and energy‐efficient preparation procedure, and competitive output power density. On the other hand, in contrast to existing self‐assembly approaches for constructing osmotic energy harvesting membranes based on synthetic polymers^[^ ^28^, ^29^, ^30^ ^]^ and crystalline porous materials,^[^ ^31^, ^32^, ^33^ ^]^ exfoliated nanosheets,^[^ ^34^, ^35^ ^]^ or extracted crystals,^[^ ^36^, ^37^ ^]^ this work demonstrates a genuine bottom‐up strategy based on small molecules and ions. This approach exemplifies the unique capability of molecular self‐assembly to hierarchically organize matter across scales—from individual molecules to nanostructures, and ultimately functional membranes. In addition, this work innovatively integrates AAO channel substrate and PVA to immobilize and stabilize the nano‐assemblies, successfully bridging the gap between molecular nano‐assemblies and functional membranes, which is expected to unlock the application potential of molecular self‐assembly to construct diverse separation membranes with tailored properties.^[^ ^64^ ^]^

Future efforts should focus on enhancing the ion selectivity and permeability of the heterogeneous membrane to further improve energy conversion efficiency and output power. The ternary molecular co‐assembly is a modular‐like design, allowing the incorporation of other polyamine compounds with higher positive charge densities as molecular modules. For instance, polyethylenimine—a versatile hydrophilic, cationic, and highly branched polymer^[^ ^65^, ^66^ ^]^—can be expected to generate a co‐assembled structure with more positive charges and more ion transport channels by forming more cross‐linking. In addition, the assembly conditions can be fine‐tuned and optimized further (e.g., by adjusting the solution to a basic pH and controlling temperature) to promote the formation of long nanofiber assemblies, through which more cross‐linked‐derived ion transport channels may be facilitated.

## Conflict of Interest

The authors declare no conflict of interest.

## Author Contributions

Y.X. and G.Q. conceived the idea. M.L. and Y.X. designed the experiments and conducted the preparation, measurements, and characterizations with assistance from F.Z., H.W., and Y.C. M.L. and X.Y. analyzed the data and wrote the manuscript under the supervision of G.Q. All authors approved the final version of the manuscript.

## Supporting information



Supporting Information

## Data Availability

The data that support the findings of this study are available from the corresponding author upon reasonable request.
