# Supplementary material for: Ternary Molecular Co‐Assembling Heterogeneous Membranes for High‐Efficiency and Anti‐Biofouling Osmotic Energy Conversion
Source: Adv Sci (Weinh). 2025 Jul 13;12(38):e04843. doi: 10.1002/advs.202504843 (PMC12520567; doi:10.1002/advs.202504843)
Supplement: Supplementary file 1 — Supporting Information [file ADVS-12-e04843-s001.docx]

Supporting Information

**Ternary Molecular** **Co-Assembling** **Heterogeneous Membranes for High-Efficiency and** **Anti-Biofouling Osmotic Energy Conversion**

Minmin Li, Yuting Xiong,* Fenglin Zhang, Haijie Wei, Yuchen Cao, and Guangyan Qing*

M. Li, Y. Xiong, F. Zhang, H. Wei, Y. Cao, and G. Qing

State Key Laboratory of Medical Proteomics, Dalian Institute of Chemical Physics, Chinese Academy of Sciences, Dalian 116023, P. R. China

Y. Xiong,

Dalian Lingshui Bay Laboratory, Dalian 116023, P. R. China

E-mail: xiongyt@dicp.ac.cn; qinggy@dicp.ac.cn.

Table of contents

[1. Materials and Instruments 3](#_Toc201860516)

[2. Experimental methods 4](#_Toc201860517)

[2.1 Fabrication of the gel membranes from the binary and ternary systems 4](#_Toc201860518)

[2.2 Fabrication of the gel membranes through addition of PVA 4](#_Toc201860519)

[2.3 Fabrication of the heterogeneous membranes 4](#_Toc201860520)

[2.4 UV-Vis and CD measurements 5](#_Toc201860521)

[2.5 SEM measurements of the solid assemblies 6](#_Toc201860522)

[2.6 DLS measurements 6](#_Toc201860523)

[2.7 Zeta potential measurements 6](#_Toc201860524)

[2.8 IR and XPS spectra measurements 7](#_Toc201860525)

[2.9 Electrical measurements 7](#_Toc201860526)

[2.10 Anti-biofouling test 8](#_Toc201860527)

[3. Energy conversion efficiency calculation 10](#_Toc201860528)

[4. Electric double layer thickness calculation 11](#_Toc201860529)

[5. Supplementary Figures and Tables 12](#_Toc201860530)

[6. References 45](#_Toc201860531)

# 1. Materials and Instruments

L-Glutathione (GSH, 99%), histamine (HA, 98%), *S*-Acetyl-L-glutathione (*S*Ac-GSH, 95%) were purchased from Aladdin Co., Ltd. (China). Silver nitrate (AgNO_3_, 99.8%), copper dinitrate (Cu(NO_3_)_2_, 98%), iron(III) chloride hexahydrate (FeCl_3_, 98%) were purchased from Sinopharm Chemical Reagent Co., Ltd. (China). Aminoguanidine (AMG, 98%), imidazole (ImD, 98%), 4-aminophenyl boronic acid (APBA, 97%), spermine (SPM, 98%), L-cysteine (Cys, 98%), *N*-acetyl-L-cysteine (*N*Ac-Cys, 98%), tetrachloroauric(III) acid trihydrate (HAuCl_4_, 99.9%) were purchased from Beijing InnoChem Co., Ltd. (China). Nanoporous anodic aluminum oxide (AAO) membranes were purchased from Hefei Puyuan nano-technology Co., Ltd. (Hefei, China). LIVE/DEAD™ BacLight™ Bacterial Viability Kits were purchased from Thermo Fisher SCIENTIFIC. Other solvents and reagents were purchased from Sinopharm Chemical Reagent Co., Ltd. (China). Ultrapure water used in this study was purified by Milli-Q system (18.2 MΩ·cm). All aqueous solutions were prepared using ultrapure water as the solvent.

UV-Vis spectra were recorded by U-3010 spectrophotometer (HITACHI Ltd., Japan). Circular dichroism (CD) spectra were recorded on a MOS-450 CD spectrometer (Bio-Logic, France). Fourier Transform Infrared Spectroscopy (IR) test was performed in the transmission mode with a resolution of 4 cm^-1^ by using Thermofisher scientific Nicolet iS50. X-ray photoelectron spectroscopy (XPS) data were obtained with a Thermo Scientific^TM^ K-Alpha^TM+^ spectrometer equipped with a monochromatic Al Kα X-ray source (1486.6 eV) operating at 100 W. Dynamic light scattering (DLS) and zeta potentials were recorded on Malvern Zetasizer Nano. Scanning electron microscope (SEM) images were recorded on Flex SEM 1000 Ⅱ and JSM 7800F (JEOL Ltd, Japan). All current-voltage curves were measured by a Keithley 6487 picoammeter (Tektronix Inc., US). Surface contact angles were recorded on Kruss DSA100. Fluorescence imaging was performed by using Olympus FV1000 MPE confocal laser scanning microscope with a microscope IX 71, a 100 × / NA 1.40 oil objective lens, LUNV series laser unit (laser combination: 405 nm; 488 nm; 543 nm; 635 nm).

# 2. Experimental methods

## 2.1 Fabrication of the gel membranes from the binary and ternary systems

For the ternary co-assembly system, a precursor solution was prepared by sequentially mixing aqueous solutions of GSH (45 mM, 5 mL), AgNO_3_ (45 mM, 5 mL) and HA (45 mM, 5 mL). Subsequently, the precursor solution was poured into a Petri dish (60 mm diameter × 15 mm depth). After about three days of assembly and solvent evaporation, the gel membrane (referred to as the GHAg membrane) was taken out from the Petri dish.

For the binary co-assembly system, a precursor solution was prepared by sequentially mixing aqueous solutions of GSH (30 mM, 5 mL) and AgNO_3_ (30 mM, 5 mL). Subsequently, the precursor solution was poured into a Petri dish (60 mm diameter × 15 mm depth). After about three days of assembly and solvent evaporation, the gel membrane (referred to as the GAg membrane) was taken out from the Petri dish.

## 2.2 Fabrication of the gel membranes through addition of PVA

For the ternary co-assembly system, a precursor solution was prepared by sequentially mixing of aqueous solutions GSH (60 mM, 5 mL), AgNO_3_ (60 mM, 5 mL), HA (60 mM, 5 mL) and PVA (2% wt, 5 mL). Subsequently, the precursor solution was poured into a Petri dish (60 mm diameter × 15 mm depth). After about three days of assembly and solvent evaporation, the membrane of GHAg was taken out from the Petri dish.

For the binary co-assembly system, a precursor solution was prepared by sequentially mixing of aqueous solutions GSH (45 mM, 5 mL), AgNO_3_ (45 mM, 5 mL), and PVA (1.5% wt, 5 mL). Subsequently, the precursor solution was poured into a Petri dish (60 mm diameter × 15 mm depth). After about three days of assembly and solvent evaporation, the membrane of GHAg was taken out from the Petri dish.

## 2.3 Fabrication of the heterogeneous membranes

First, the AAO membranes were processed with oxygen plasma at 200 W for 10 minutes to generate hydroxyl groups and make them hydrophilic. After rinsing with excess ultrapure water and ethanol, the PAA membrane was dried at room temperature. Then a cleaned AAO membrane was first secured to the Petri dish bottom by adhering its four corners with adhesive tape, as shown in Figure S6. Then, a precursor solution was prepared by sequentially mixing aqueous solutions of GSH (60 mM, 5 mL), AgNO_3_ (60 mM, 5 mL), HA (60 mM, 5 mL) and PVA (2% wt, 5 mL). Subsequently, the precursor solution was poured into the Petri dish with an AAO membrane fixed on the bottom. Gently shake the dish to ensure that the solution can enter the nanopores by capillary action from the top. After three days of assembly and solvent evaporation in an ambient environment (21~23 °C of temperature, 40%~60% of humidity), the AAO membrane with a layer of gel covered was taken out from the Petri dish. The heterogeneous membrane, AAO@GHAg/PVA, was obtained after carefully peeling off the upper gel layer and rinsing with ultrapure water.

A similar method was also used to prepare the AAO@GAg/PVA membrane. A precursor solution was prepared by sequentially mixing of GSH (45 mM, 5 mL), AgNO_3_ (45 mM, 5 mL), and PVA (1.5% wt, 5 mL) aqueous solutions.

This heterogeneous membrane fabrication approach was also applied to prepare control membranes, including: the AAO@GHAg membrane (without PVA), the AAO@GH/PVA membrane (without Ag⁺ ions), and a series of variant membranes produced by systematically modifying individual components (as shown in Figure 6 of the main text). The concentrations of each component in each precursor solution are all referenced to the concentrations of the above-mentioned three-component or two-component precursor solutions. Moreover, it is worth noting that for those precursor solutions that cannot form assemblies, the resulting heterogeneous membrane should be called the deposited membrane.

## 2.4 UV-Vis and CD measurements

For the binary system, a series of solutions were prepared by continuously adding different volumes of AgNO_3_ solution (10 mM) (i.e., 0, 3, 6, 9, 12, 15, 18, 21, and 24 μL) to the stock solution of GSH (10 mM, 30 μL) and further diluting to 3 mL with ultrapure water. Then, UV-Vis and CD spectra of these solutions were recorded sequentially.

Similarly, for the ternary system, 15μL of GSH (20 mM) and 15μL of HA (20 mM) was mixed to offer a stock solution. Then a series of solutions were prepared by continuously adding different volumes of AgNO_3_ (10 mM) (i.e., 0, 3, 6, 9, 12, 15, 18, 21, and 24 μL) to the stock solution containing GSH and HA and further diluting to 3 mL with ultrapure water. Then, UV-Vis and CD spectra of these solutions were recorded sequentially.

The same method as mentioned above is also used in UV-Vis test of other systems depicted in Figure 6 in the main text.

## 2.5 SEM measurements of the solid assemblies

First, a mixture solution containing equal concentration (1 mM) of GSH and AgNO_3_ was prepared and left overnight to produce the solid assemblies. Then 10 μL of the dispersion solution containing solid assemblies was dropped onto a cleaned silicon wafer, followed by natural evaporation to obtain a dry sample. And their SEM images could be obtained after spray gold processing.

Similarly, for the ternary co-assembly system, the mixture solution containing equal concentration (1mM) of GSH, HA, and AgNO_3_ was prepared. The other steps are the same as above.

## 2.6 DLS measurements

First, for the binary co-assembly system, a mixture solution containing equal concentration (0.1 mM) of GSH and AgNO_3_ was prepared and left overnight. For the ternary co-assembly system, a mixture solution containing equal concentration (0.1 mM) of GSH, HA, and AgNO_3_ was prepared and left overnight. Then the resulting mixture solutions were subjected to the DLS measurement.

## 2.7 Zeta potential measurements

The solid membranes (including the cleaned AAO substrate and other heterogeneous membranes) were carefully ground in an agate mortar for approximately 30 minutes to obtain a fine, homogeneous powder. This extended grinding ensured a uniform particle size distribution while minimizing aggregation, which is essential for subsequent characterization. The resulting powder could then be effectively dispersed in ultrapure water (pH 6.5) at a concentration of 0.5 mg/mL. The zeta potential of the powder suspension was measured using the Malvern Zetasizer Nano. All tests were performed in ultrapure water at neutral pH (6.5) without additional pH adjustment, to ensure that the conditions of the test suspension are consistent with those of the solution during the osmotic energy harvesting evaluation.

## 2.8 IR and XPS spectra measurements

An equimolar (1.5 mM each) aqueous mixture of GSH, AgNO_3_, and HA was first prepared and allowed to react for 3 days to form solid assemblies (i.e., the GHAg assemblies). The resulting precipitates were collected by centrifugation (8,000 rpm, 10 min), followed by freeze-drying for subsequent IR and XPS characterization.

## 2.9 Electrical measurements

The osmotic energy conversion property of the heterogeneous membrane was evaluated by performing the transmembrane ionic current measurement in the form of the current−voltage (*I*−*V*) test by using a picoammeter (Keithley 6487, Tektronix, Inc.). The heterogeneous membrane was mounted between two compartments of a home-made electrochemical device by using two silicon wafers with holes in the center as gaskets on both sides of the membrane. Both silicon wafers have a square pore with a side length of approximately 0.065 mm. In this case, the effective test membrane area is 0.0043 mm^2^. Each compartment was filled with 2 mL of salt solution as the electrolyte solution. A pair of Ag/AgCl electrodes with AgCl-coated Ag wires with 0.5 mm in diameter immersed in saturated KCl solutions as salt bridges were selected to carry out the *I*−*V* test. Then, the *I*−*V* data was recorded under sweep voltages varying from −0.2 V to +0.2 V with a step voltage of 0.02 V and a step time of 1 second. Each test was repeated at least three times to obtain the average current value at different voltages. After finishing a test, both electrolyte solutions in two compartments were replaced with fresh solutions. On the performance stability test, the heterogeneous membrane that was mounted in the electrochemical device stays submerged in the electrolyte solution for the entire duration of the test. The electrolyte solutions in both compartments need to be replaced with fresh solutions before each measurement. In addition, to investigate the effect of the testing membrane area, in addition to the gaskets we used (aperture area: 0.0043 mm^2^), the other two gaskets with aperture areas of 0.0314, and 1.1304 mm^2^ were also employed.

Seawater from the Yellow Sea and freshwater from Dalian Xishan Lake as river water were used to create a natural seawater/river water system. Prior to use, these waters are first filtered through medium-speed filter paper to remove sediment, floating substances, and so on. The *I*−*V* test was then carried out by adding these two types of water to the reservoirs of the electrochemical device.

The temperature-dependent osmotic energy harvesting performance was systematically evaluated using a drying oven for thermal control. Prior to measurement, both the NaCl solutions (stored in sealed centrifuge tubes to prevent evaporation) and the membrane-equipped electrochemical device were pre-heated and equilibrated to the target temperature (approximately 30 min). We then added the preheated solutions to the electrochemical device and started recording data once the current had stabilized. The solution and device were then preheated together to the next target temperature. Add the preheated solution to the device to replace the original solution, rinse two or three times and then run a test.

## 2.10 Anti-biofouling test

First, gram-negative *E. coli* and gram-positive *S. aureus* were chosen as two indicator bacteria. The bacteria were cultivated in Luria-Bertani (LB) culture medium under shaking (200 rpm, 15 h) at 37°C to reach the growth phase. After incubation, the bacteria were harvested by centrifuged at 4000 rpm for 5 min and re-suspended in sterile PBS. The final concentration of bacteria solutions was adjusted to OD600nm to 0.1.

The AAO@GHAg/PVA membrane was sterilized by UV irradiation for 1 hour. Then, the membrane samples with 1 cm × 1 cm were immersed in 12-well plate containing 1 mL of bacteria solution and incubated at 37 °C for 12 h. After incubation, the samples were rinsed with 5 mL of ultrapure water and then submerged in a beaker with 10 mL of phosphate-buffered saline (PBS). Sonication was performed for about 3 minutes before collecting the resulting wash solution. A 50 μL aliquot of the wash solution was evenly spread on Luria-Bertani (LB) agar plates and incubated under identical temperature conditions for 12 h.^[1]^

For comparison purposes, the pristine AAO membrane samples underwent identical incubation conditions with bacterial suspension as controls. The number of colonies formed was counted to determine the antimicrobial efficacy (*E*_a_).^[2]^

$$\begin{aligned} E_{a}\left( \% \right)=\frac{N_{AAO}-N_{n}}{N_{AAO}}\times100 \#\left( 1 \right) \end{aligned}$$

Additionally, the osmotic energy conversion performance of the AAO@GHAg/PVA membrane was assessed after bacterial adhesion to determine the impact of its antifouling properties.

**Bacteria observation by fluorescence microscopy**: The bacteria solution of *E. coli* was added to a 12-well plate containing samples and incubated at 37 °C for 12 h. Afterward, the unattached bacteria on samples were removed by neutral PBS buffer. Samples were stained by LIVE/DEAD™ BacLight™ Bacterial Viability Kits. Fluorescence microscopy was used to observe samples.^[3]^

**Bacteria observation by scanning electronic microscopy**: The samples with the size of 1 cm × 1 cm were cocultured with *E. coli* for 12 h. Then, all the samples were cleaned with PBS and treated with 2.5% Glutaraldehyde EM Grade (Solarbio, China) at 4 °C overnight. A graded series of alcohol (30%, 50%, 75%, 85%, 95%, and 100%, respectively) was applied for sample dehydration for 15 min each. The dehydrated samples were dried and subsequently coated with gold. Morphology observations were under SEM with the exposure parameters of 20 kV.^[4]^

# 3. Energy conversion efficiency calculation

The cation transference number (*t*_+_) that quantifies the ion selectivity can be calculated by Nernst equation:^[5]^

$$\begin{aligned} 2t_{+}=\frac{V_{OC}}{\frac{RT}{zF}\ln\left( \frac{\gamma_{H}C_{H}}{\gamma_{L}C_{L}} \right)}+1 \#\left( 2 \right) \end{aligned}$$

$$\begin{aligned} t_{-}=1-t_{+} \#\left( 3 \right) \end{aligned}$$

Where *R*, *T*, *z*, *F*, *γ* and *C* refer to the gas constant, temperature, ion valence, Faraday constant, activity coefficient, and salt concentration, respectively. If *t*_+_ > 0.5 indicates the membrane with cation selectivity and *t*_+_ < 0.5 indicates the membrane with anion selectivity.

Upon obtaining *t*_+_, the energy conversion efficiency (*η*) corresponding to the maximum power can be determined by

$$\begin{aligned} \eta=\frac{\left( 2t_{+}-1 \right)^{2}}{2}\times100\% \#\left( 4 \right) \end{aligned}$$

The transference number of cations as well as conversion efficiency of the AAO@GHAg/PVA as a function of the NaCl gradient were summarized in Table S1.

#

# 4. Electric double layer thickness calculation

The electric electrical double layer thickness is also called the Debye length (λ_D_), which can be calculated using the formula:^[6]^

$$\begin{aligned} \lambda_{D}=\sqrt{\frac{\varepsilon_{r}\varepsilon_{0}k_{B}T}{2e^{2}C}}\#\left( 5 \right) \end{aligned}$$

Where $\varepsilon_{r}$, $\varepsilon_{0}$, $k_{B}$, *T*, *e*, and *C* refers to the relative permittivity of water, vacuum permittivity, Boltzmann constant, temperature, elementary charge, and ionic concentration, respectively.

# 5. Supplementary Figures and Tables


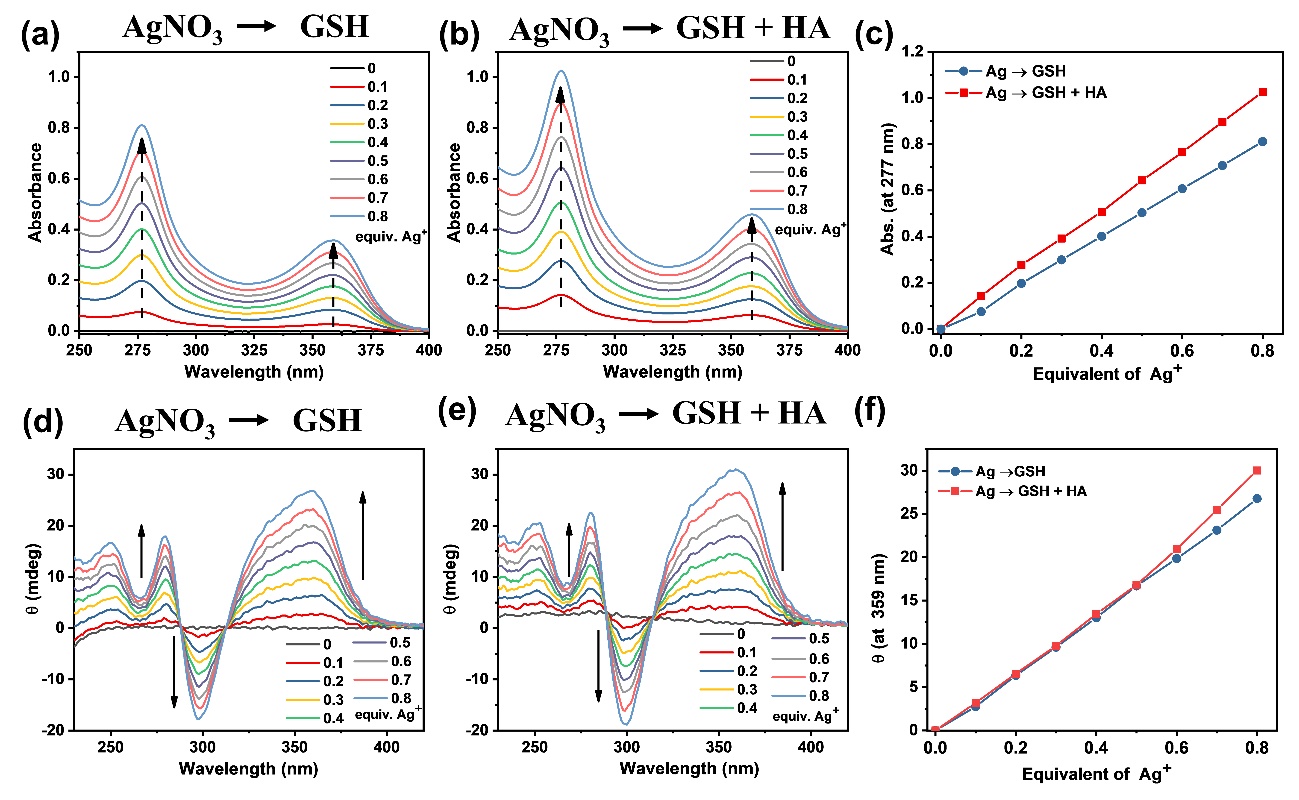


**Figure S1**. UV-Vis spectra (a, b) and CD spectra (c, d) of GSH solution (a, c) and the mixed solution of GSH and HA (b, d) after adding different molar ratios of AgNO_3_ solution. Comparison of the intensity increase (at 277 nm) in UV-Vis spectra (e) and the change in θ (at 359 nm) in CD spectra (f) between the ternary and binary systems.


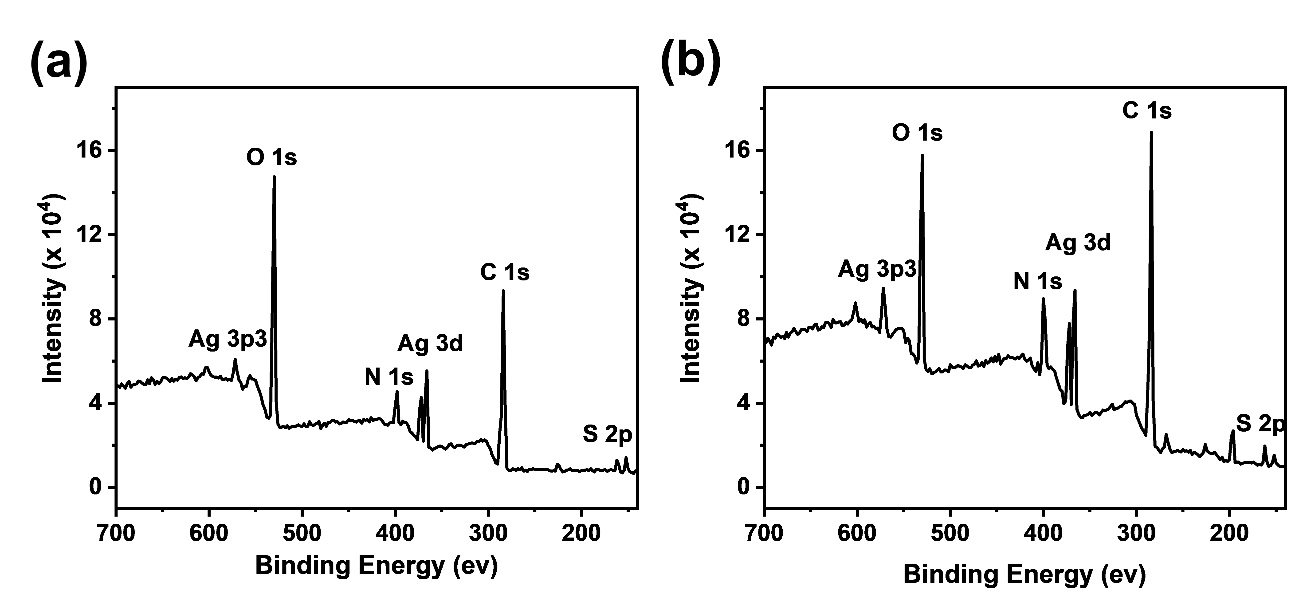


**Figure S2**. X-ray photoelectron spectroscopy (XPS) wide-scan spectra of the GAg assemblies from the binary system (a) and the GHAg assemblies from the ternary system (b).


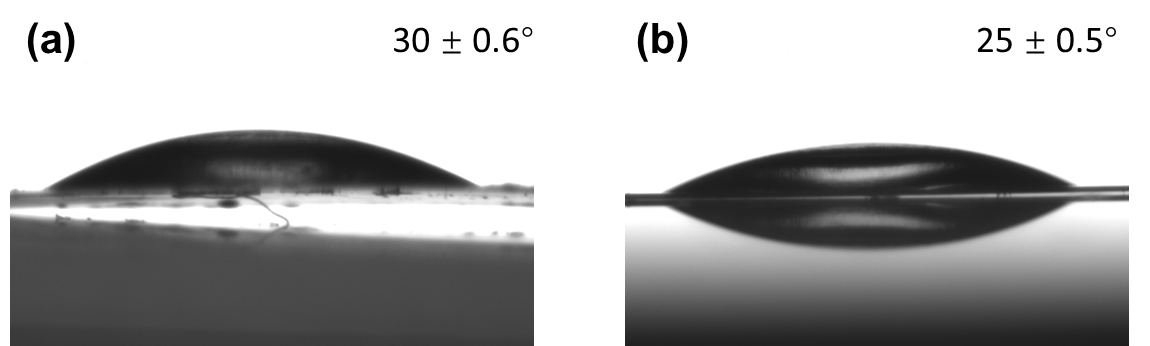


**Figure S3**. Surface water contact angle of the GAg/PVA gel membrane (a) and the GHAg/PVA gel membrane (b).


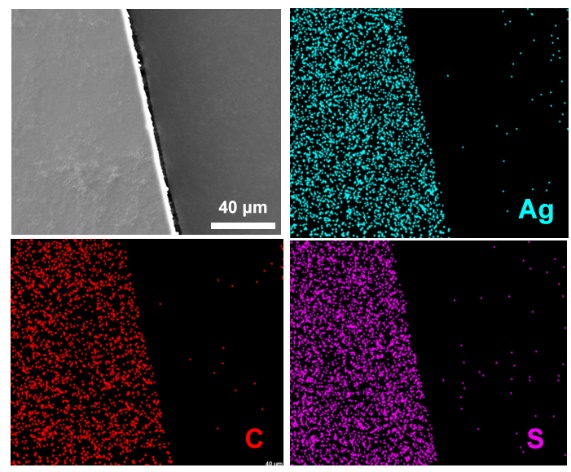


**Figure S4**. SEM image and the corresponding EDX elemental mappings of Ag, C and S of the GAg/PVA gel membrane.


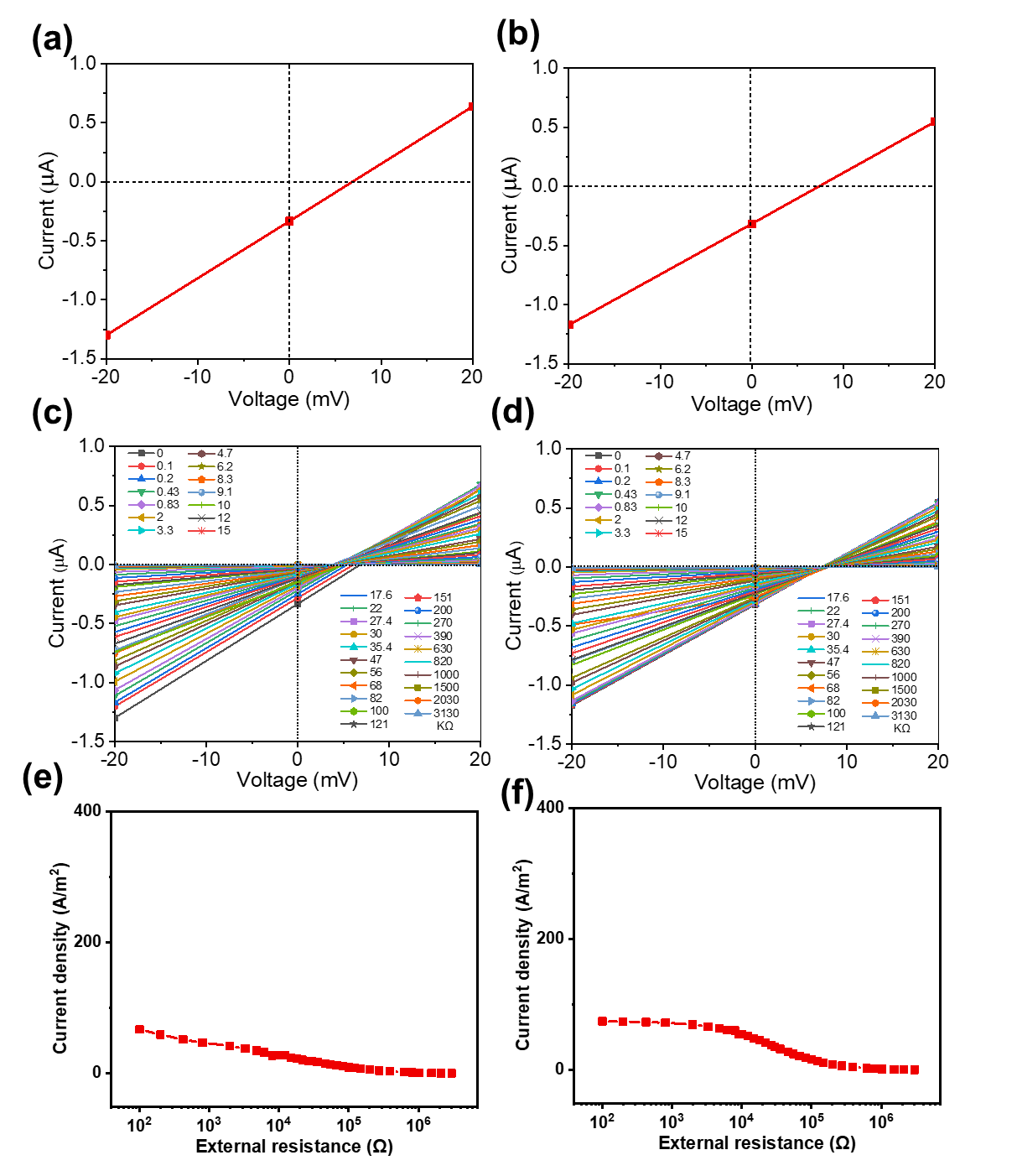


**Figure S5**. (a,b) *I–V* curves of GAg/PVA (a) and GHAg/PVA (b) under a 50-fold NaCl gradient (0.5 M NaCl *versus* 10 mM NaCl). (c,d) *I–V* curves of GAg/PVA (c) and GHAg/PVA (d) under varying external loads and a fixed 50-fold NaCl gradient. (e,f) Output current density *versus* external resistance of GAg/PVA (e) and GHAg/PVA (f) membranes under a 50-fold NaCl gradient.


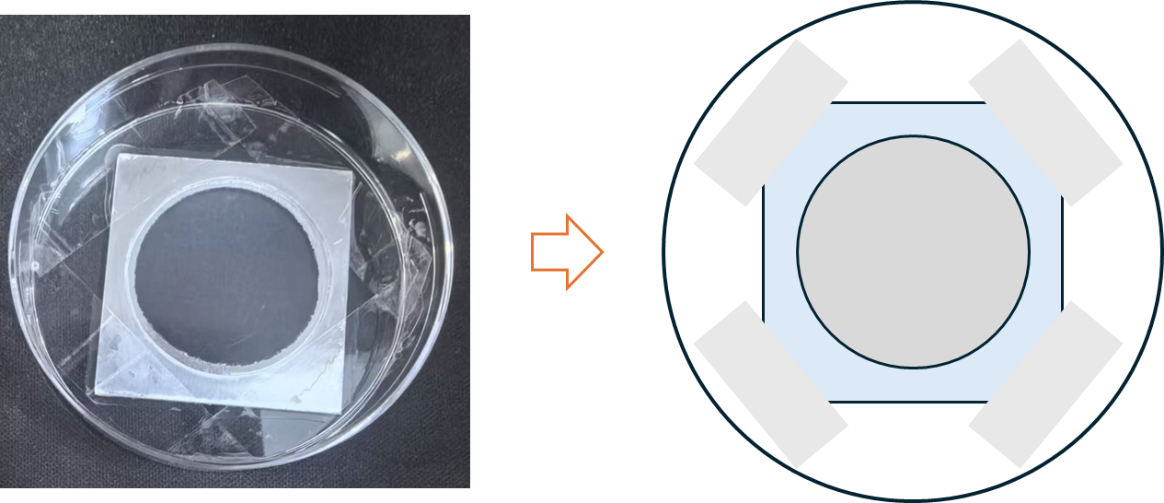


**Figure S6**. The AAO membrane was secured to the Petri dish bottom by adhering its four corners with adhesive tape.


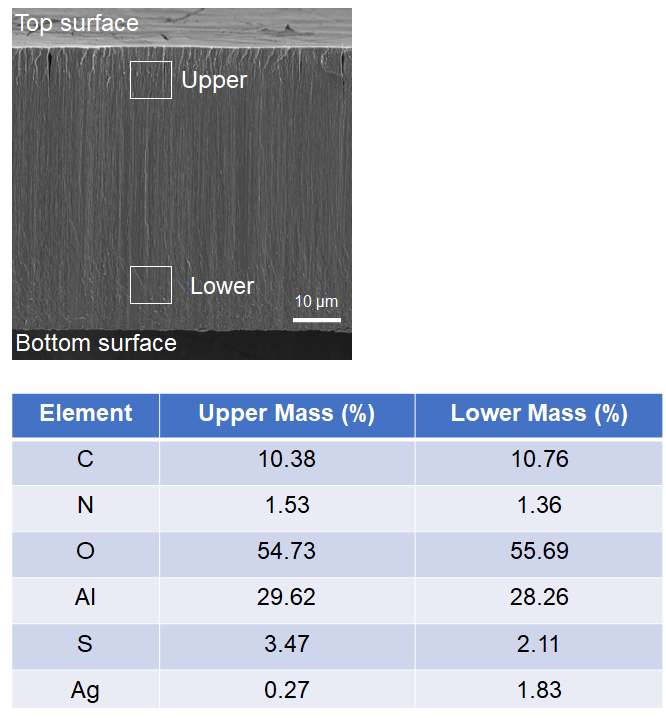


**Figure S7**. EDX elemental analysis of the upper and lower cross-sections of the AAO@GHAg/PVA heterogeneous membrane.


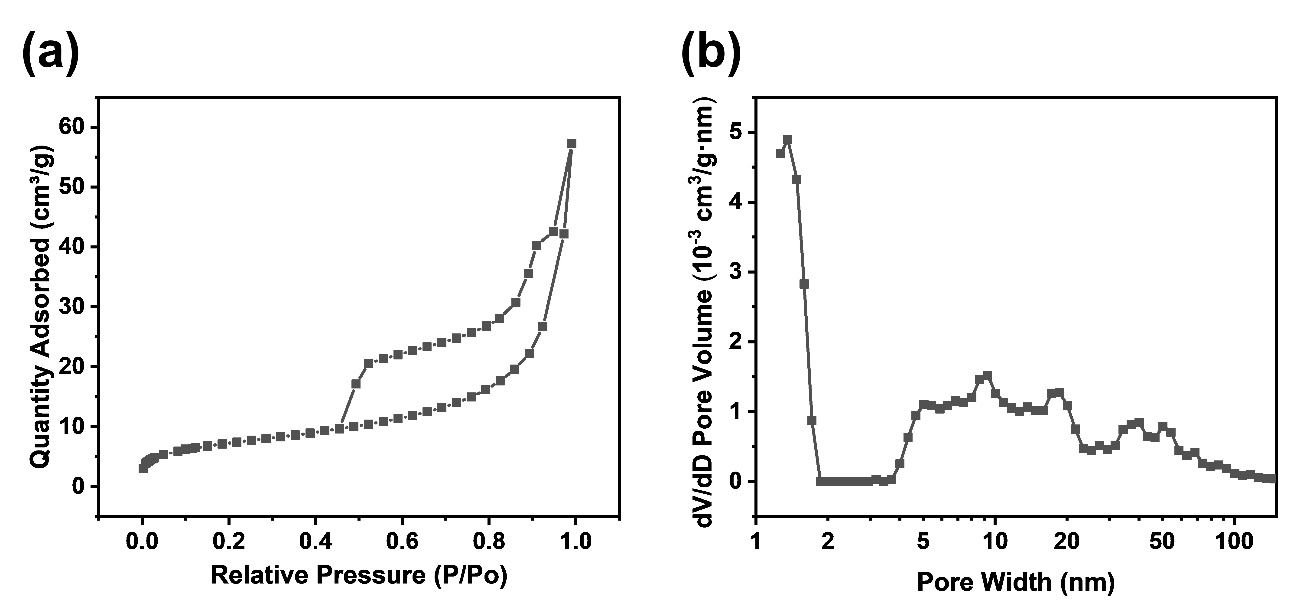


**Figure S8**. BET analysis of AAO@GHAg/PVA. (a) N_2_ adsorption-desorption isotherms. (b) Corresponding pore size distribution curve.


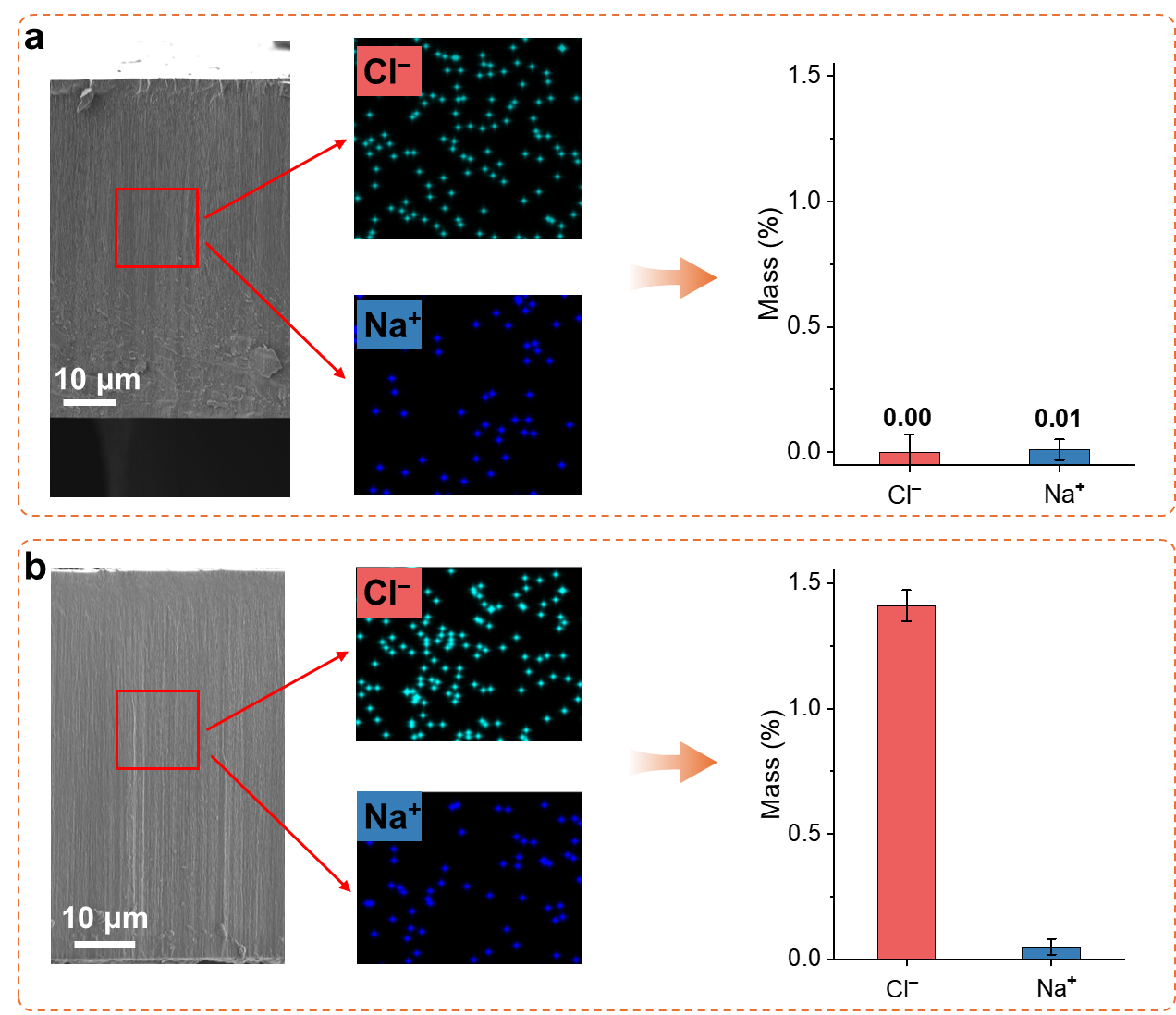


**Figure S9**. Ion selectivity investigation of AAO@GHAg/PVA heterogeneous membrane using EDX elemental analysis. (a) Untreated, freshly prepared AAO@GHAg/PVA membrane. (b) AAO@GHAg/PVA membrane following overnight immersion in 1 M NaCl solution.


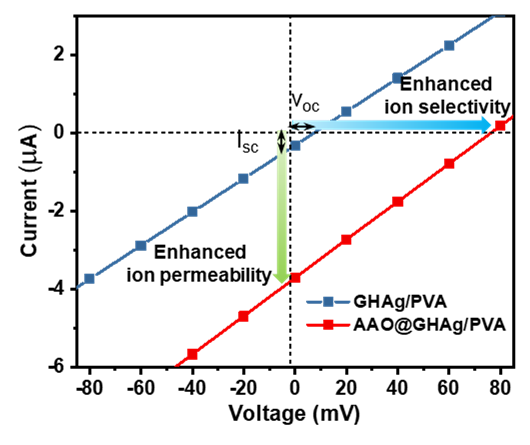


**Figure S10**. Comparison of *I–V* curves of the GHAg/PVA gel membrane and the AAO@GHAg/PVA heterogeneous membrane under a 50-fold NaCl gradient.


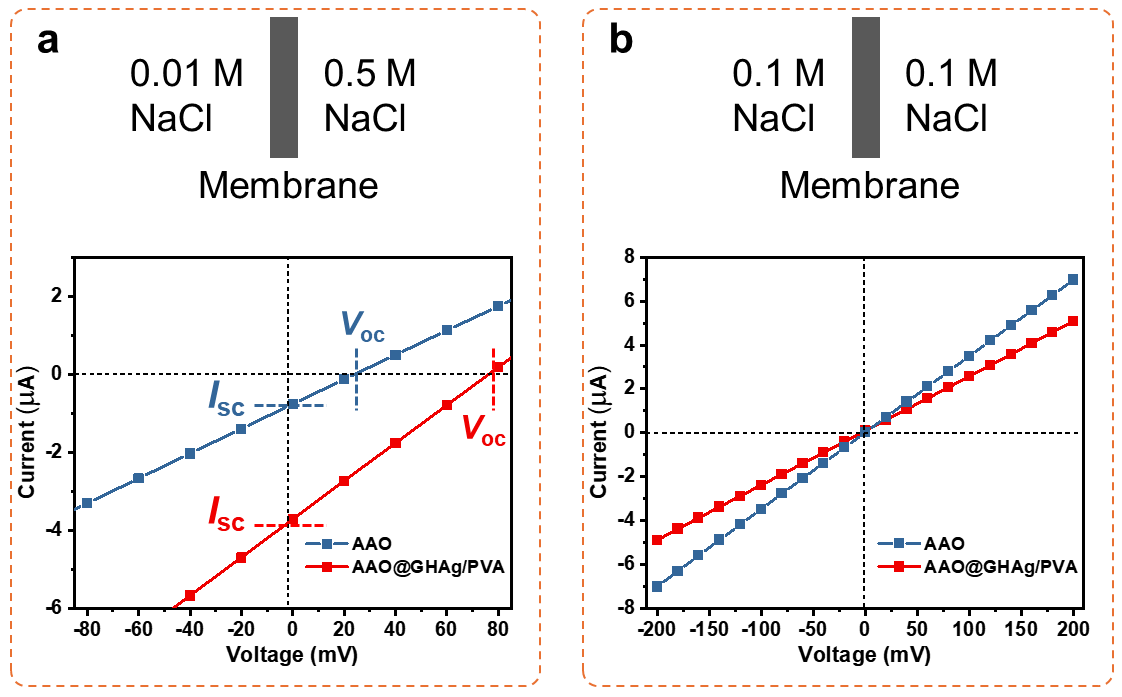


**Figure S11**. Comparison of *I–V* curves of the AAO@GHAg/PVA heterogeneous membrane and the pristine AAO membrane under (a) a 50-fold NaCl gradient and (b) symmetric 0.1 M NaCl conditions.


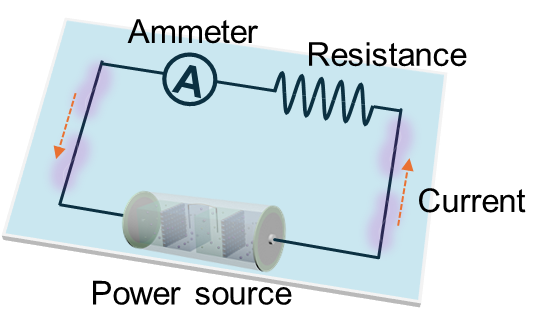


**Figure S12**. The equivalent circuit incorporating an adjustable external resistance for evaluating osmotic energy harvesting performance.


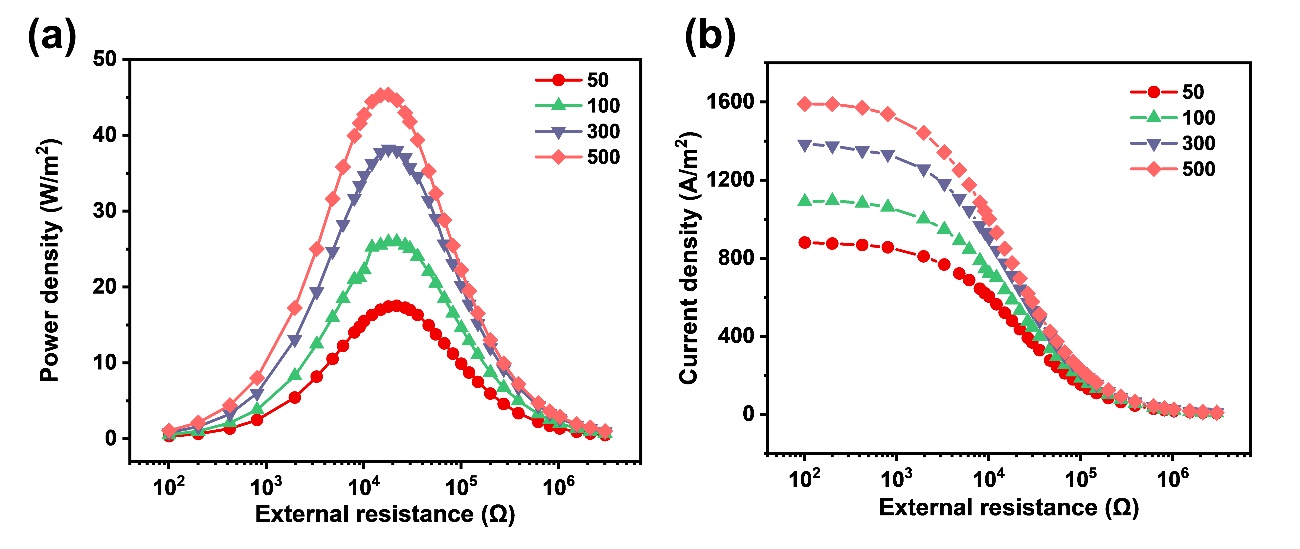


**Figure S13**. Output power density (a) and current density (b) of the AAO@GHAg/PVA membrane as functions of external resistance, measured across various NaCl concentration gradients (with fixed 10 mM low-concentration side).


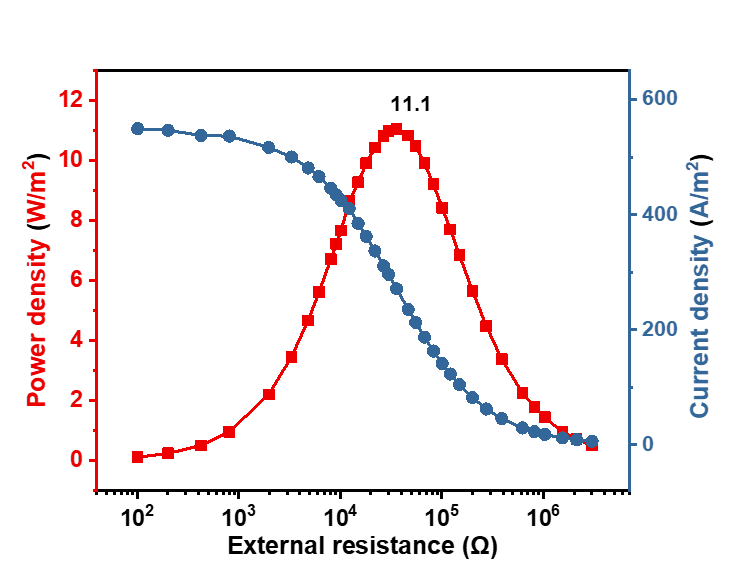


**Figure S14**. Output power density and current density of the AAO@GAg/PVA membrane as functions of external resistance, measured under a 50-fold NaCl gradient.


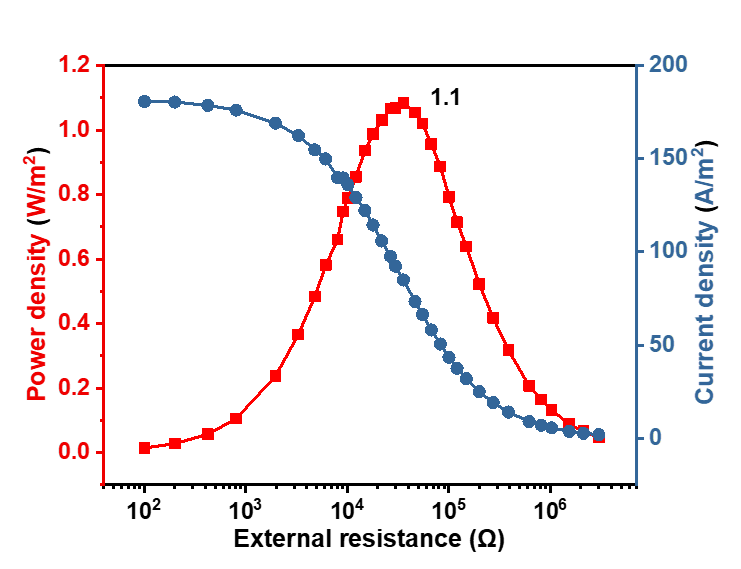


**Figure S15**. Output power density and current density of the pristine AAO membrane as functions of external resistance, measured under a 50-fold NaCl gradient.


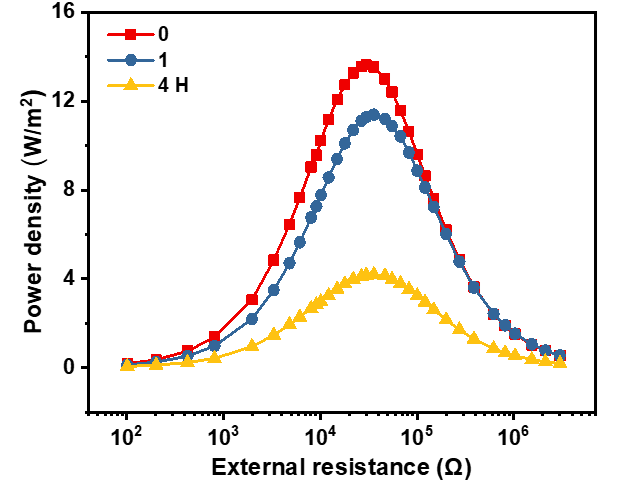


**Figure S16**. Output power density versus external resistance for a PVA-free heterogeneous membrane (AAO@GHAg), measured over a 4-hour testing period.


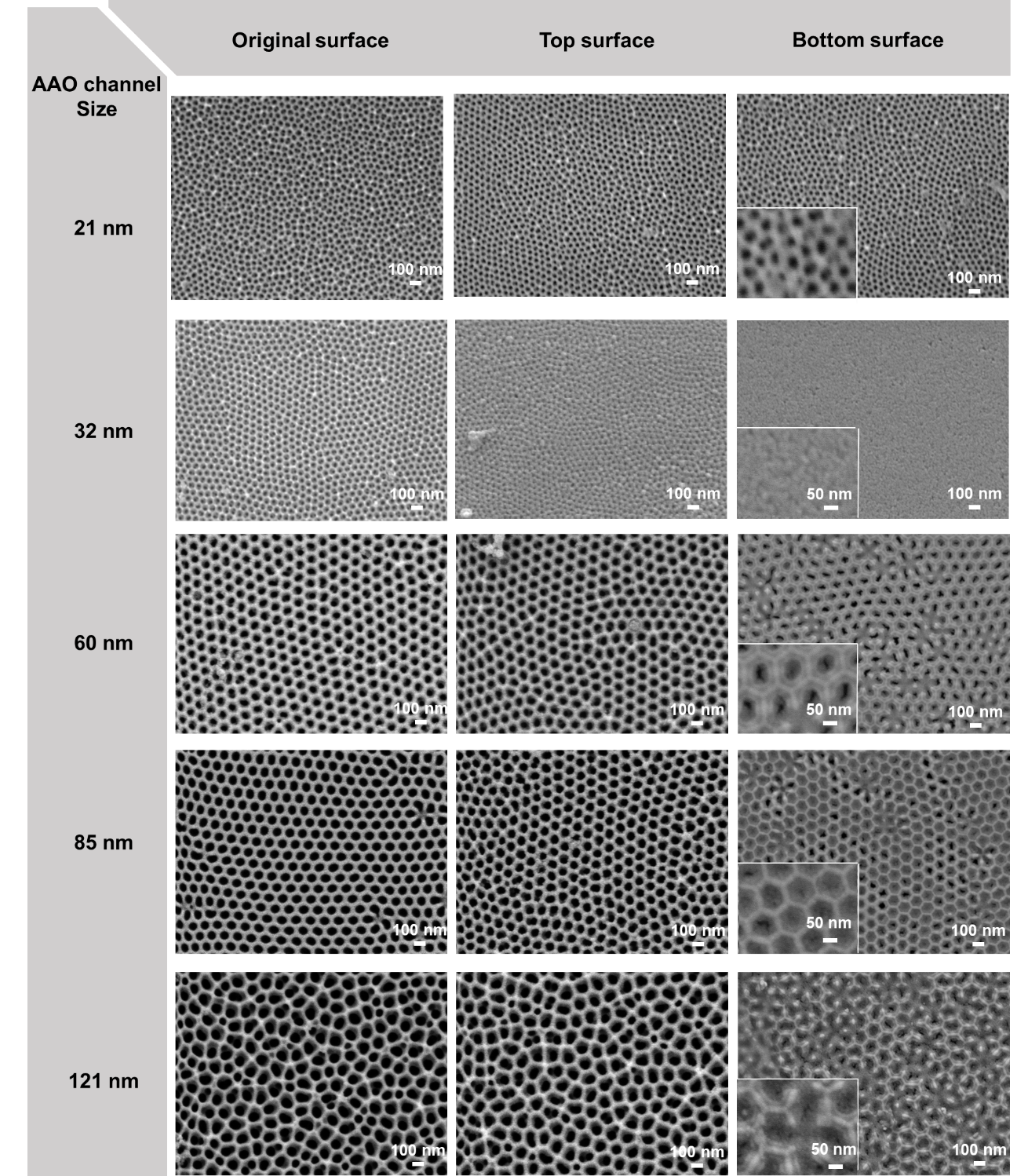


**Figure S17**. SEM images showing the pristine AAO substrates with varying mean pore diameters (left column) and the top (middle column) and bottom (right column) surfaces of corresponding AAO@GHAg/PVA heterogeneous membranes.


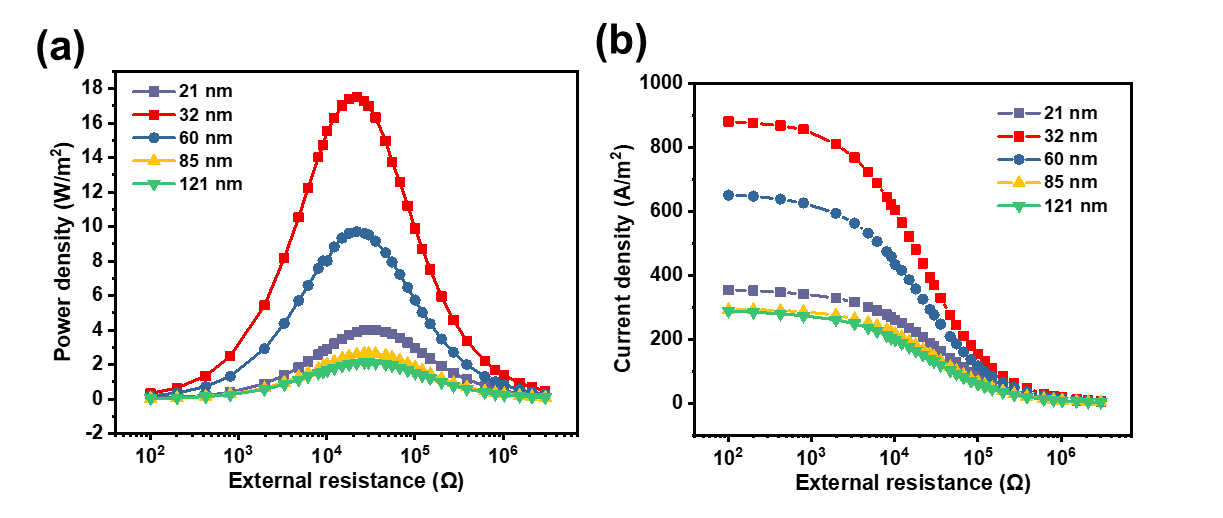


**Figure S18**. Output power (a) and current density (b) as functions of external resistance for the AAO@GHAg/PVA membranes fabricated from AAO substrates with different pore diameters, measured under a 50-fold NaCl gradient.


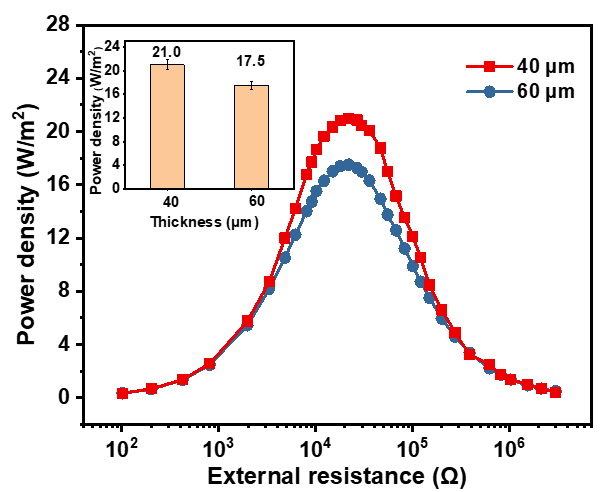


**Figure S19**. Comparison of output power densities between 40 μm- and 60 μm-thick AAO@GHAg/PVA membranes (channel diameter: 30-40 nm). The inset shows their peak output power densities.


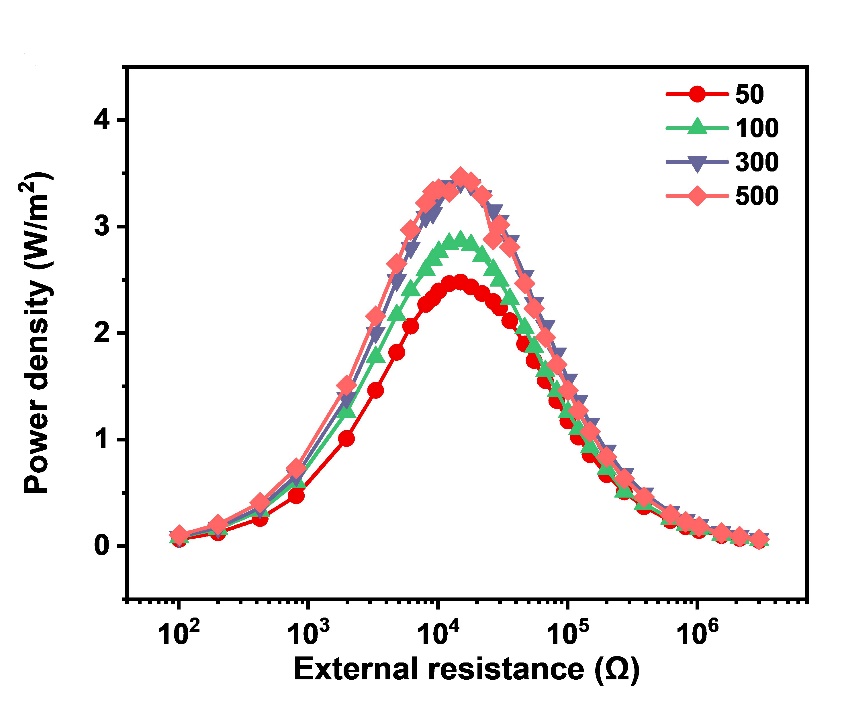


**Figure S20**. Output power densities of an AAO@GHAg/PVA membrane measured under different NaCl concentration gradients, with an effective testing area of 0.0314 mm^2^.

**
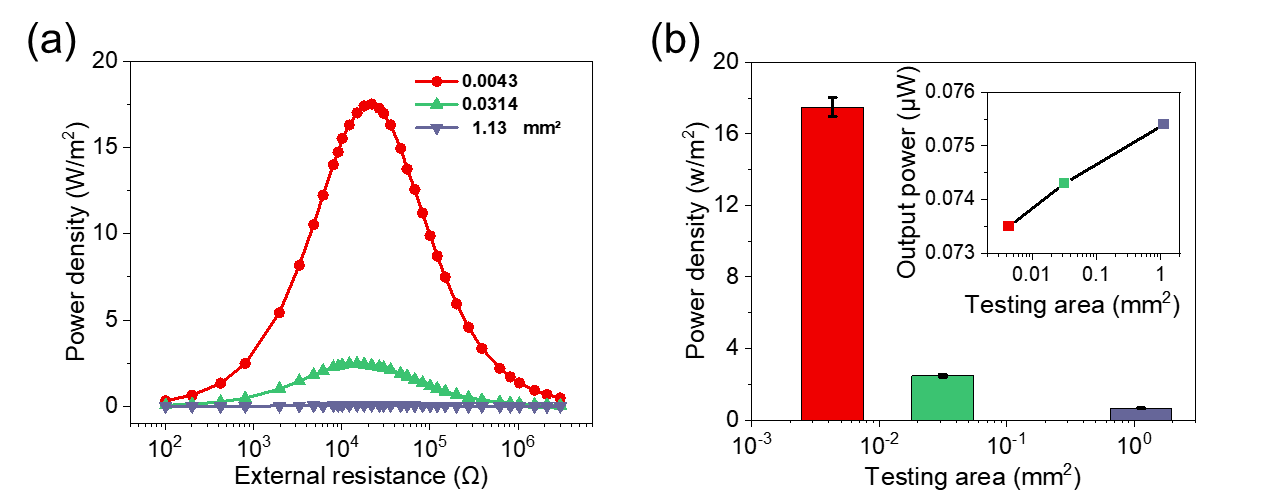
**

**Figure S21**. (a) Output power densities of the AAO@GHAg/PVA measured with different testing area under a 50-fold NaCl gradient. (b) The corresponding peak output power density. Inset is the total output power.


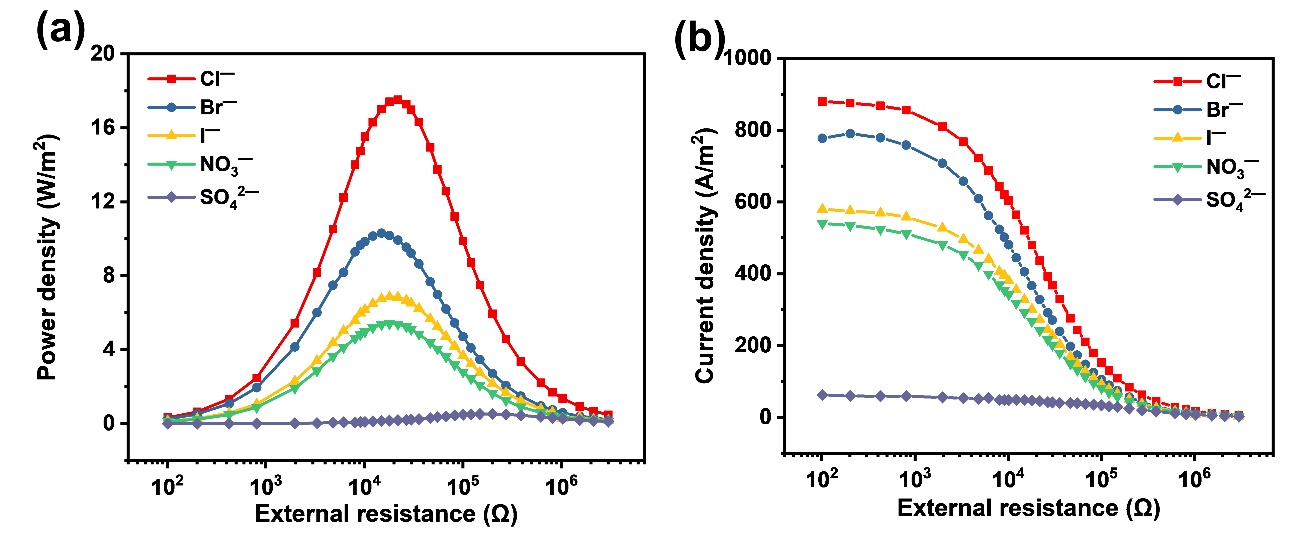


**Figure S22**. Output power (a) and current density (b) as functions of external resistance for an AAO@GHAg/PVA membrane, measured under a 50-fold salinity gradient toward different anions.


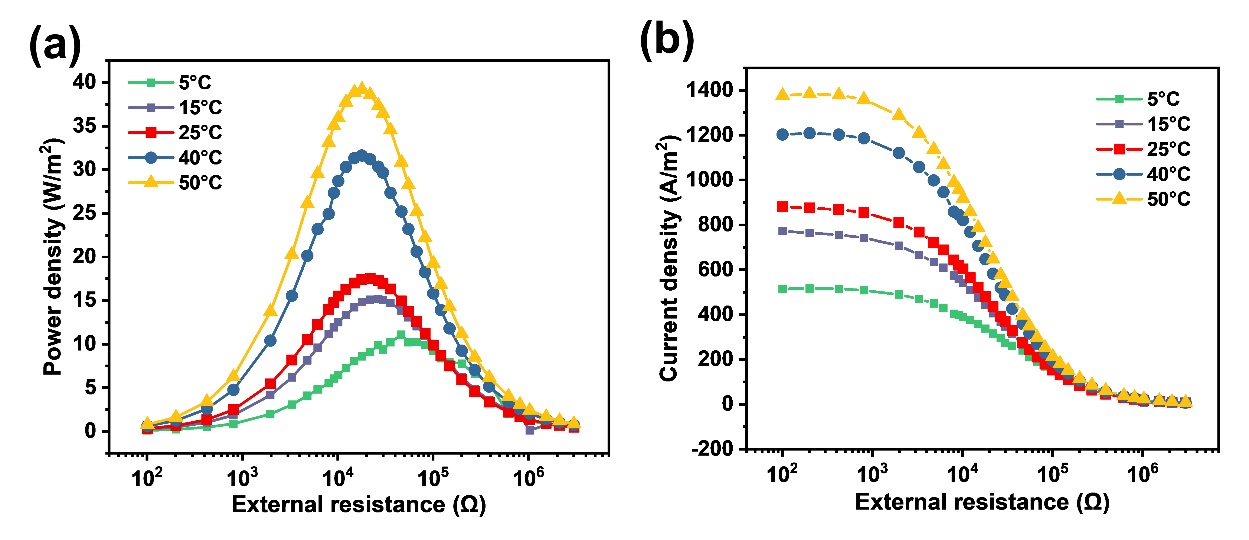


**Figure S23**. Output power (a) and current density (b) as functions of external resistance for an AAO@GHAg/PVA membrane, measured under a 50-fold NaCl gradient at different temperatures.


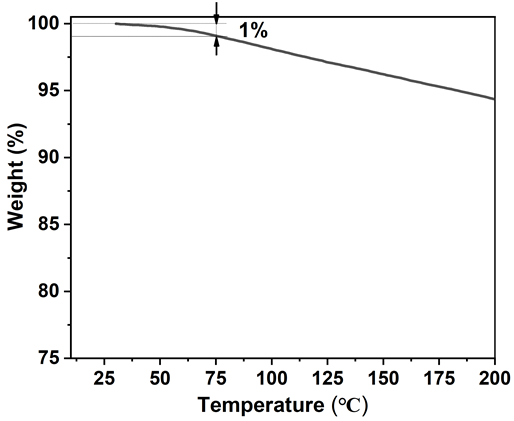


**Figure S24**. Thermo gravimetric analysis (TGA) curve of the AAO@GHAg/PVA membrane.


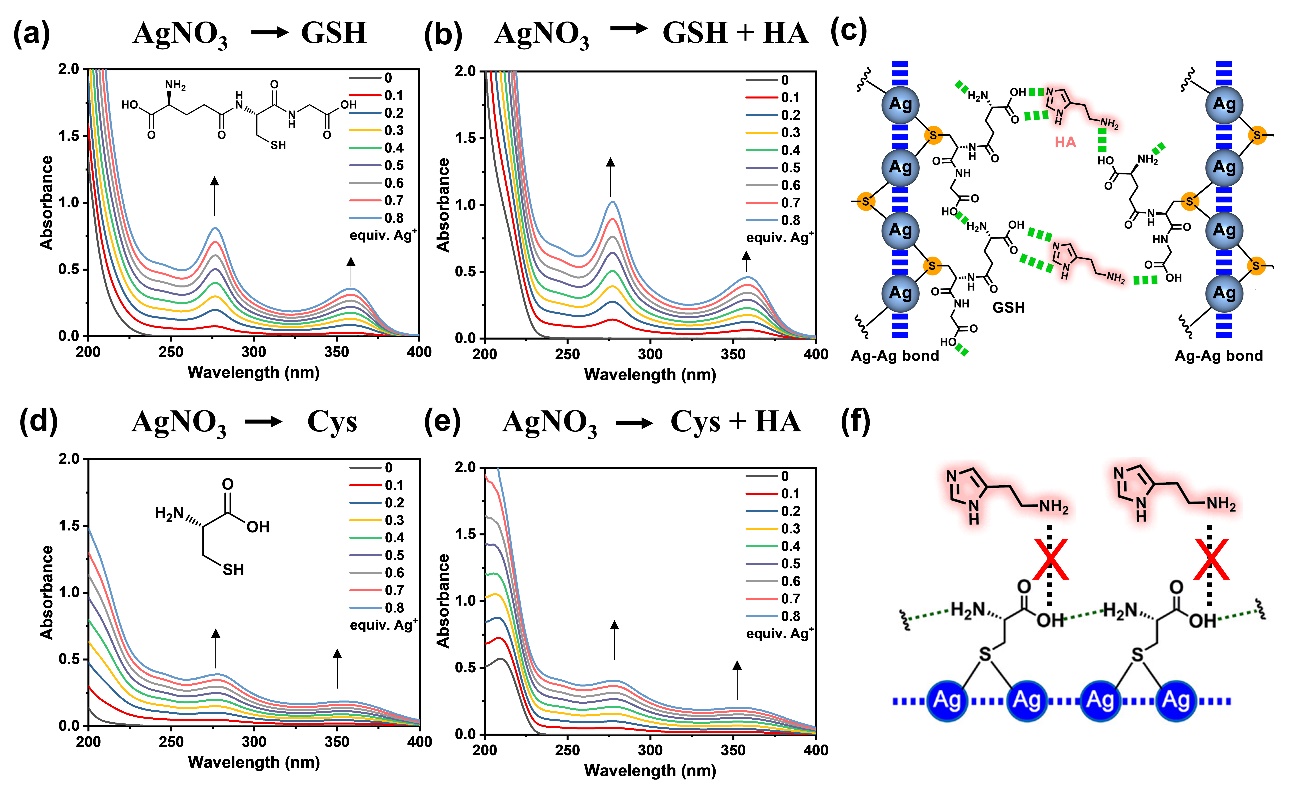


**Figure S25**. UV-Vis of GSH solution (a) and the mixed solution of GSH and HA (b) after adding different molar ratios of AgNO_3_ solution. (c) Schematic illustrating the possible coordination and complexation. UV-Vis of Cys solution (d) and the mixed solution of Cys and HA (e) after adding different molar ratios of AgNO_3_ solution. (f) Schematic illustrating the hydrogen bonding interactions cannot be formed.


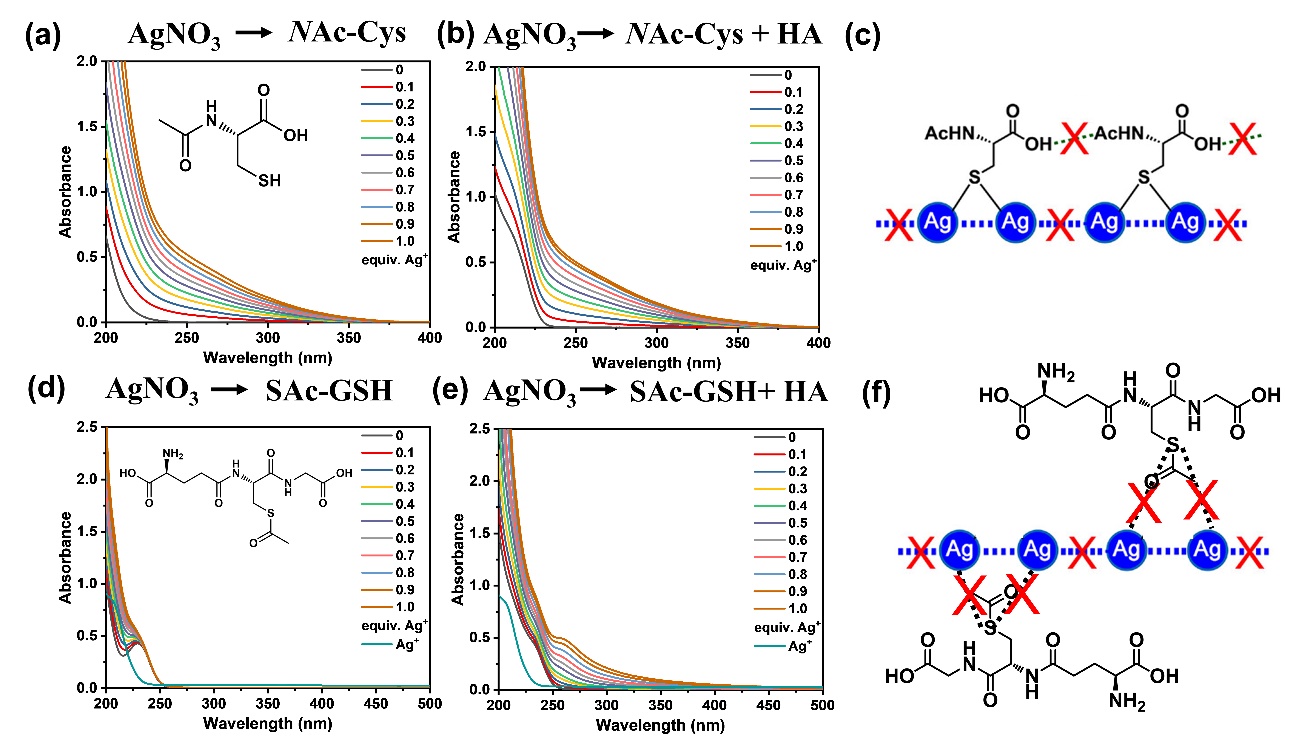


**Figure S26**. UV-Vis of *N*Ac-Cys solution (a) and the mixed solution of *N*Ac-Cys and HA (b) after adding different molar ratios of AgNO_3_ solution. (c) Schematic illustrating the coordination or complexation interactions cannot be formed. UV-Vis of *S*Ac-Cys solution (d) and the mixed solution of *S*Ac-Cys and HA (e) after adding different molar ratios of AgNO_3_ solution. (f) Schematic illustrating the coordination or complexation interactions cannot be formed.


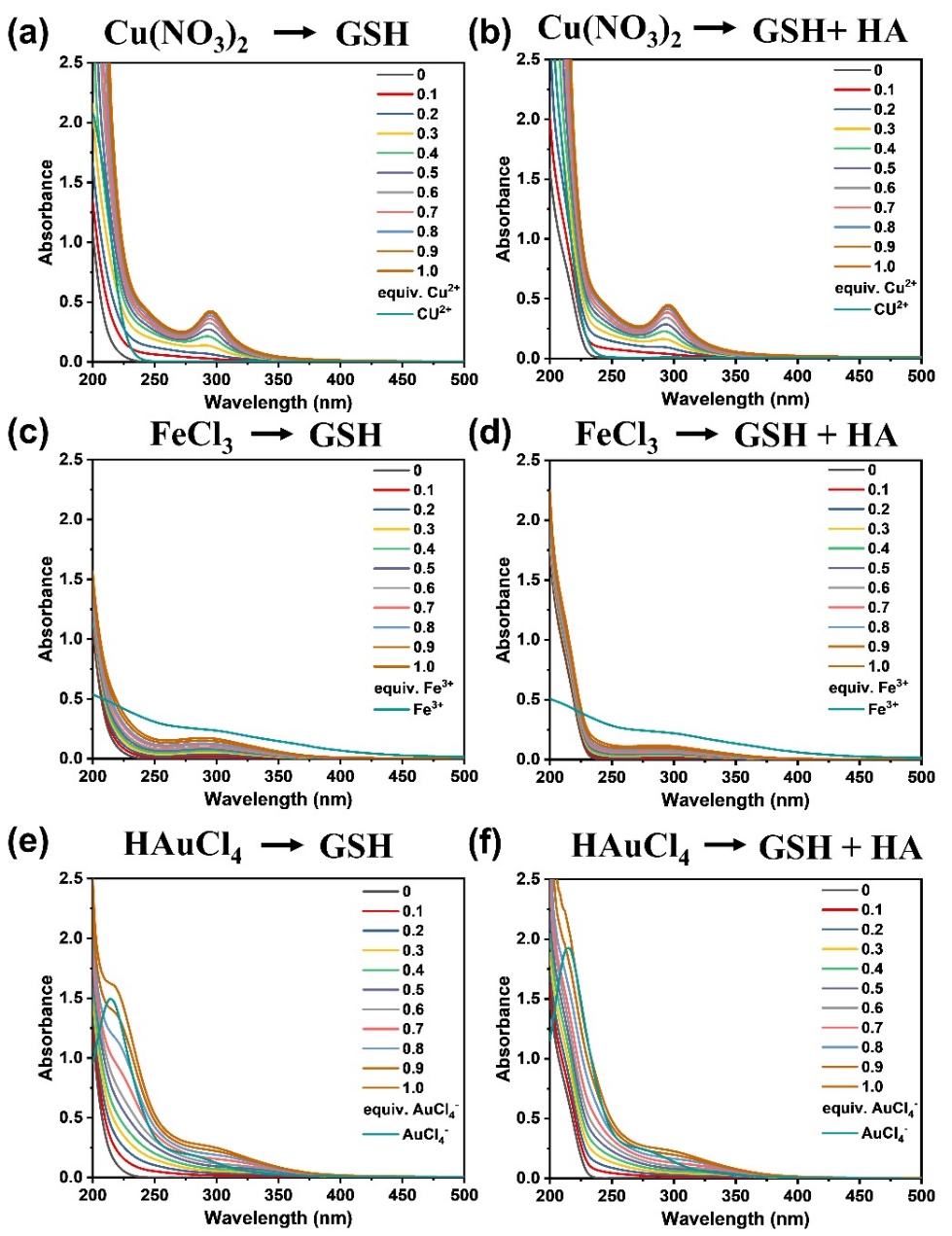


**Figure S27**. UV-Vis of GSH solutions (a, c, e) and the mixed solutions of GSH and HA solution (b, d, f) after adding different equivalent of Cu(NO_3_)_2_ (a, b), FeCl_3_ (c, d) and HAuCl_4_(e, f) solution.

**Comments**: -SH-containing compounds (e.g., GSH here) can coordinate with Cu^2+^ ions to form assemblies.^[7]^ However, the actual assembly is usually constrained by solution conditions, such as concentration, pH, and temperature, etc. Under the assembly conditions here, Cu^2+^ ions failed to coordinate with GSH due to the neutral solution condition (requiring alkaline conditions).

According to Hard and Soft Acid and Base (HSAB) theory, Fe^3+^ is a hard acid, while thiol (-S^−^) is a soft base, and their coordination is relatively poor (Hard acids are more inclined to coordinate with hard bases such as O/N). Moreover, Fe^3+^ ions and thiol compounds are prone to oxidation-reduction reactions, which leads to the consumption of thiol compounds.

The interaction between [AuCl_4_]^−^ and SH-containing compounds involves complex chemistry. Au^3+^ (a soft acid) in [AuCl_4_]^−^ preferentially binds with -SH (a soft base). As a result, Cl⁻ ligands are gradually replaced with -S^−^. Moreover, SH-containing compounds can also reduce Au^3+^ to Au^+^ or Au^0^. These competing reactions deplete available thiol groups, leaving insufficient -SH to form stable -S^−^-Au^+^ coordination complexes required for subsequent assembly.

Overall, in our experimental setup using equimolar GSH and metal ions (and HA) in aqueous solution, GSH was unable to coordinate with Cu^2+^ ions, Fe^3+^ ions, and [AuCl_4_]^−^ ions, preventing assembly formation.

~~

~~

**Figure S28**. Output current and power density as functions of external resistance for the Ag^+^-free AAO@GH/PVA membrane, measured under a 50-fold NaCl gradient.


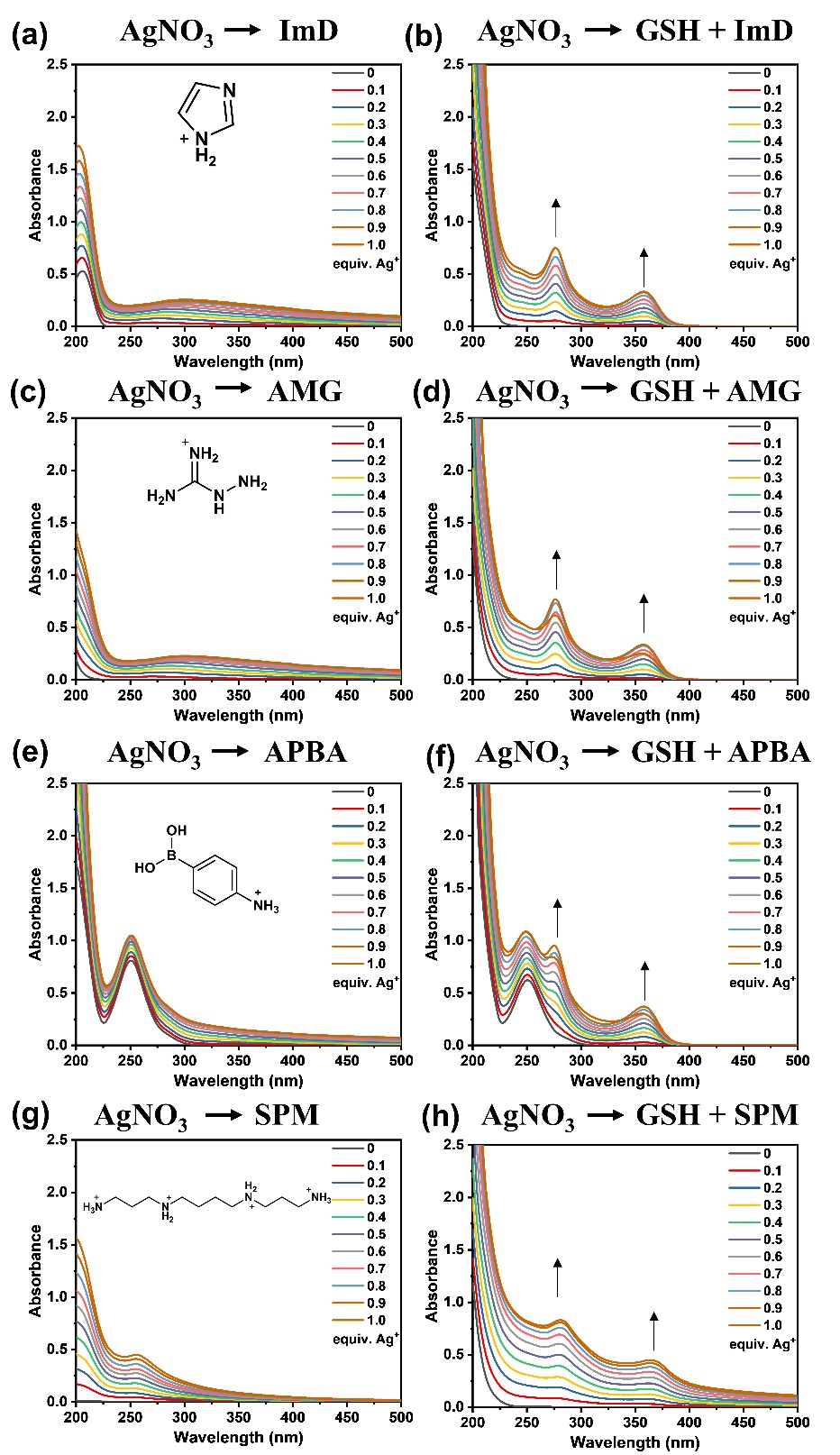


**Figure S29**. UV-Vis of ImD solution (a) and the mixed solution of GSH and ImD (b), AMG solution (c) and the mixed solution of GSH and AMG (d), APBA solution (e) and the mixed solution of GSH and APBA (f), SPM solution (g) and the mixed solution of GSH and SPM (h), after adding different equivalent of AgNO_3_ solution.

**
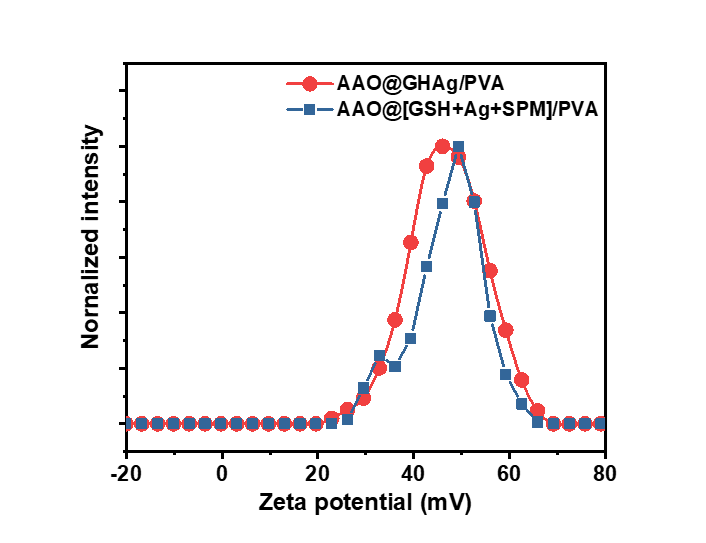
**

**Figure S30**. Comparison of zeta potential data between the AAO@GHAg/PVA membrane and the AAO@[GSH+Ag+SPM]/PVA membrane. The zeta potential was measured by grinding the membrane into a fine powder and dispersing it in water.

**
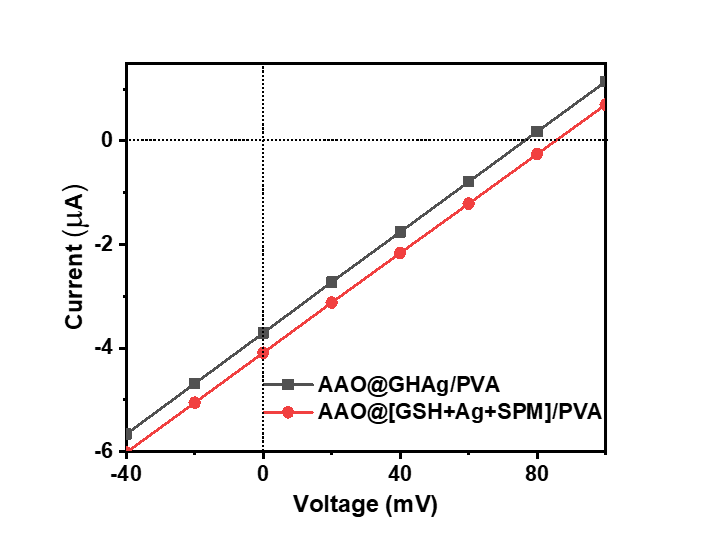
**

**Figure S31**. Comparison of *I−V* curves between the AAO@GHAg/PVA membrane and the AAO@[GSH+Ag+SPM]/PVA membrane measured under a 50-fold NaCl gradient.

**Table S1**. *V*_oc_, *t*_−_, and 𝜂 of the AAO@GHAg/PVA membrane at different NaCl gradients.

| Concentration gradient  (mM/mM) | *V*_oc_(mV) | *t*_−_ | 𝜂(%) |
| --- | --- | --- | --- |
| 10/1 | 36.4 | 0.81 | 19.0 |
| 50/1 | 62.5 | 0.807 | 19.3 |
| 100/1 | 73.1 | 0.809 | 19.1 |
| 500/1 | 100.3 | 0.814 | 19.7 |
| 1000/1 | 107.7 | 0.803 | 18.4 |
| 5000/1 | 103.2 | 0.736 | 11.1 |

**Table S2**. Electrical double layer thickness ($\lambda_{D}$) of nanochannels of the AAO@GHAg/PVA membrane in different NaCl concentration solutions.

| Concentration  (mM) | *λ_D_*  (nm) |
| --- | --- |
| 10 | 3.04 |
| 50 | 1.36 |
| 100 | 0.96 |
| 500 | 0.43 |
| 1000 | 0.30 |
| 5000 | 0.14 |

# 6. References

[1] Q. Yu, Y. Yuan, J. Wen, X. Zhao, S. Zhao, D. Wang, C. Li, X. Wang, N. Wang, *Adv. Sci.* **2019**, *6*, 1900002.

[2] H. Wang, Y. Zhang, J. Wang, Saijilahu, H. Sun, H. Yang, X.-H. Xia, C. Wang, *Adv. Funct. Mater.* **2025**, *35*, 2412477.

[3] K.-T. Huang, W.-H. Hung, Y.-C. Su, F.-C. Tang, L. D. Linh, C.-J. Huang, L.-H. Yeh, *Adv. Funct. Mater.* **2023**, *33*, 2211316.

[4] Y. Tang, K. Wang, B. Wu, K. Yao, S. Feng, X. Zhou, L. Xiang, *Adv. Mater.* **2024**, *36*, 2307756.

[5] J. Yang, B. Tu, G. Zhang, P. Liu, K. Hu, J. Wang, Z. Yan, Z. Huang, M. Fang, J. Hou, Q. Fang, X. Qiu, L. Li, Z. Tang, *Nat. Nanotechnol.* **2022**, *17*, 622.

[6] Z.-Q. Li, G.-L. Zhu, R.-J. Mo, M.-Y. Wu, X.-L. Ding, L.-Q. Huang, Z.-Q. Wu, X.-H. Xia, *Angew. Chem. Int. Ed.* **2022**, *61*, e202202698.

[7] B. Ma, S. Wang, F. Liu, S. Zhang, J. Duan, Z. Li, Y. Kong, Y. Sang, H. Liu, W. Bu, L. Li, *J. Am. Chem. Soc.* **2019**, *141*, 849.
